# The establishment of Central American migratory corridors and the biogeographic origins of seasonally dry tropical forests in Mexico

**DOI:** 10.3389/fgene.2014.00433

**Published:** 2014-12-19

**Authors:** Charles G. Willis, Brian F. Franzone, Zhenxiang Xi, Charles C. Davis

**Affiliations:** ^1^Center for the Environment, Harvard UniversityCambridge, MA, USA; ^2^Department of Organismic and Evolutionary Biology, Harvard University HerbariaCambridge, MA, USA

**Keywords:** adaptive lag time, diversification, land bridge, long-distance dispersal, pre-adaptation, tropical biogeography, South America, species pool

## Abstract

Biogeography and community ecology can mutually illuminate the formation of a regional species pool or biome. Here, we apply phylogenetic methods to a large and diverse plant clade, Malpighiaceae, to characterize the formation of its species pool in Mexico, and its occupancy of the seasonally dry tropical forest (SDTF) biome that occurs there. We find that the ~162 species of Mexican Malpighiaceae represent ~33 dispersals from South America beginning in the Eocene and continuing until the Pliocene (~46.4–3.8 Myr). Furthermore, dispersal rates between South America and Mexico show a significant six-fold increase during the mid-Miocene (~23.9 Myr). We hypothesize that this increase marked the availability of Central America as an important corridor for Neotropical plant migration. We additionally demonstrate that this high rate of dispersal contributed substantially more to the phylogenetic diversity of Malpighiaceae in Mexico than *in situ* diversification. Finally, we show that most lineages arrived in Mexico pre-adapted with regard to one key SDTF trait, total annual precipitation. In contrast, these lineages adapted to a second key trait, precipitation seasonality, *in situ* as mountain building in the region gave rise to the abiotic parameters of extant SDTF. The timing of this *in situ* adaptation to seasonal precipitation suggests that SDTF likely originated its modern characteristics by the late Oligocene, but was geographically more restricted until its expansion in the mid-Miocene. These results highlight the complex interplay of dispersal, adaptation, and *in situ* diversification in the formation of tropical biomes. Our results additionally demonstrate that these processes are not static, and their relevance can change markedly over evolutionary time. This has important implications for understanding the origin of SDTF in Mexico, but also for understanding the temporal and spatial origin of biomes and regional species pools more broadly.

## Introduction

The application of phylogenetics has stimulated the field of community ecology (Webb et al., 2002; Cavender-Bares et al., 2009). Beyond informing us on the nature of community assembly in the present, however, phylogenetic community ecology also holds the promise of integrating deep evolutionary history to understand the origin of communities in relation to geographic and climatological changes across tens of millions of years (Emerson and Gillespie, 2008). In this spirit, we envision the eventual merger between the fields of “community ecology” and “biogeography.” More specifically, we imagine a time in the near future where every species within a community can be placed into its broader phylogenetic context, allowing us to pinpoint each species time and place of origin, and its broader pattern of trait evolution and diversification. From an ecological perspective, biogeographic approaches can provide insight into the origin of the larger species pool for a given region. And from a biogeographic perspective, community ecology can provide insight into the ecological processes that structure and maintain diversity within the same region.

In biogeographic studies, a common way to delineate the formation of a species pool is to focus on the larger geographic region or a particular biome within that region. A major topic along these lines, but one that has not been sufficiently treated for most biomes and taxa, involves the role of migration (originating *ex situ*) vs. diversification (originating *in situ*) in shaping a regional biota (Emerson and Gillespie, 2008). These processes are not mutually exclusive, of course, but represent ends of a spectrum. Yet, the extent to which either of these processes dominates remains poorly understood. Classic island biogeography predicts that migration will dominate in regions that are both new or near a source species pool, while diversification will dominate in regions that are old or isolated (MacArthur and Wilson, 1967; Emerson and Gillespie, 2008; Losos and Ricklefs, 2009). An example of the former is the origination of *Oxalis* diversity in the Atacama Desert, where the modern *Oxalis* species pool was formed through multiple dispersals into the Atacama Desert region from geographically adjacent, likely pre-adapted lineages (Heibl and Renner, 2012). In contrast, the remarkable species diversity in the Andes is often attributed to prolific *in situ* diversification (Bell and Donoghue, 2005; Hughes and Eastwood, 2006; Antonelli et al., 2009). The Andean uplift and the development of more temperate environments effectively isolated this region from its surrounding tropical species pool (i.e., “continental island” effect) and permitted the colonization of temperate lineages via a small number of dispersals events (often only one from within a single larger clade), which subsequently radiated. Understanding how the balance between dispersal and diversification for a given biome changes with time, however, remains poorly understood.

A major component necessary for understanding the balance between migration and diversification is the degree to which lineages are pre-adapted to newly inhabited regions (Donoghue, 2008). Along these lines, it has been hypothesized that given the prevalence of phylogenetic niche conservatism in plants, species may more frequently migrate into regions to which they are pre-adapted, i.e., “it is easier to move than to evolve” (Donoghue, 2008). For instance, lineages from a clade that share a pre-adaption to a new biome are more likely to establish there, in which case the biome's species pool is likely to be assembled through multiple dispersal events. In contrast, lineages from a clade that are not pre-adapted will be required to adapt subsequent to their arrival to successfully establish. To the extent that *in situ* adaptation is difficult, lineages that disperse into the biome will fail to establish, thus favoring *in situ* diversification from a smaller number of dispersals. Perhaps the most striking evidence of the former scenario was presented by Crisp et al. (2009), who identified a broad pattern of phylogenetic bias in the tendency for lineages to disperse and establish across ecologically similar biomes.

**Figure 1:**
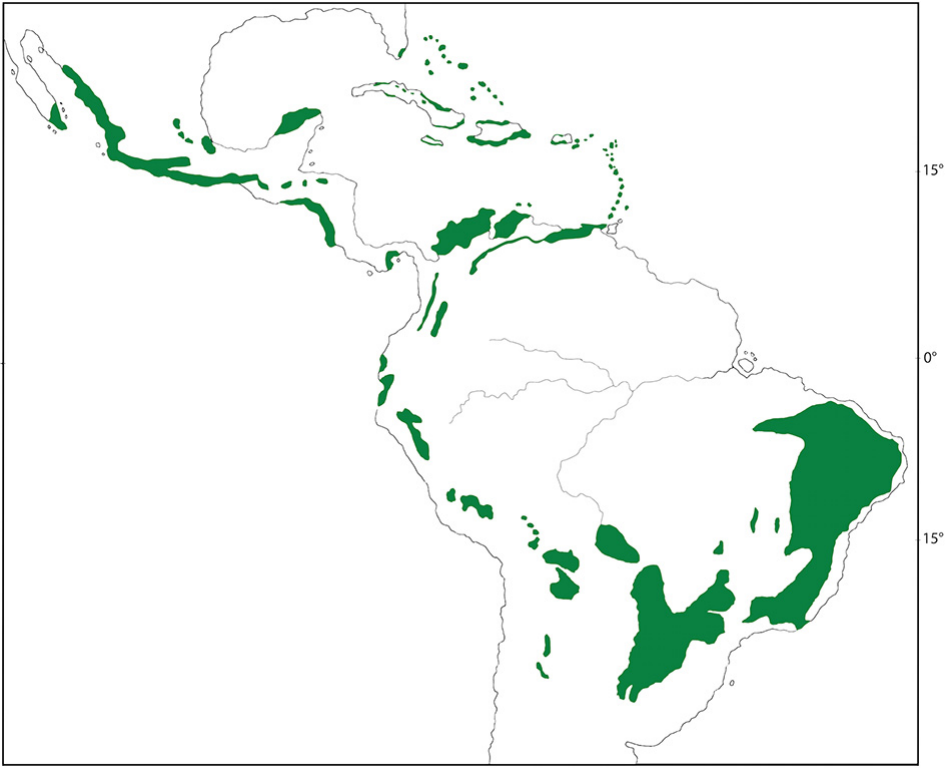
**Distribution of seasonal dry tropical forest (SDTF) across the New World (green)**. Distribution map is modified from Pennington et al. (2006b).

Short of a complete phylogeny for both the biome and its resident species, it is most effective at this stage to focus on a representative clade that forms an important component of a biome's diversity. Here, we investigate a biome of high diversity, seasonally dry tropical forest (SDTF) in Mexico, and a clade of flowering plants, Malpighiaceae, that represents an important component of this diversity. Mexico is one of the top ten megadiverse countries in the world and contains a variety of tropical biomes (Williams et al., 2001). Among its most prominent and distinct biomes is the widespread SDTF, which blankets the Pacific slopes of Mexico, ranging from central Sonora and southeastern Chihuahua to the southern state of Chiapas southward into Central America (Figure 1). SDTF is determined by precipitation patterns characterized by total annual precipitation less than 1800 mm yr^−1^ and a distinct seasonally dry period with less than 100 mm over 5–6 months (Murphy and Lugo, 1986; Gentry, 1995). At 1800 mm yr^−1^, SDTF falls below the threshold of what defines a tropical rainforest (2000 mm yr^−1^). What further distinguishes SDTF is their striking seasonality in precipitation, which results in a distinctly green period for half of the year, followed by an equally long dry period during which many species lose their leaves. In terms of their physiognomy, Mexican SDTF is characterized by low to medium trees in which grasses are a minor component (Pennington et al., 2000).

Details on the origin of the Mexican SDTF flora, and the timing of its expansion, have received recent attention (reviewed in De-Nova et al., 2012). The challenge of understanding the origin and expansion of this biome is attributed to scant fossil evidence, as well as, complex geological changes that created the conditions necessary for the biome's characteristic climate (Graham, 2010). Geologically, the establishment of the SDTF in Mexico likely began with the advent of mountain building in southern Mexico during the late Eocene. Today, the north-south Sierra Madre Occidental and the east-west Neovolcanic mountain chains greatly maintain the climatic conditions of this biome, especially by blocking cold fronts from the north. The last uplift of the Sierra Madre Occidental was between 34 and 15 Myr, while the Neovolcanic mountain chain was thought to have been established more recently and in several stages, from west to east, beginning ~23 Myr and ending only recently, 2.5 Myr. Thus, the formation of SDTF in Mexico likely occurred slowly and steadily over a period spanning at least 34 Myr, coincident with these major mountain building events. Becerra (2005) used a time-calibrated phylogeny of the prominent Mexican dry forest clade *Bursera* to assess the origin and expansion of the SDTF flora. Her findings indicated that the oldest Mexican *Busera* began to diversify between 30 and 20 Myr in western Mexico, in concert with the origination of the Sierra Madre Occidental. In contrast, younger lineages (>17 Myr) diversified in south-central Mexico, with the expansion of the east-west Neovolcanic mountain chain. These results suggest that SDTF formation was greatly facilitated by mountain building in this region and that this biome first established in western Mexico during the Oligocene and subsequently expanded south and east in Mexico, and eventually to Central America (Graham and Dilcher, 1995; Becerra, 2005).

Malpighiaceae are a pantropical clade and include ~1300 species, 90% of which are found in the New World (Davis et al., 2001; Anderson et al., 2006 onwards; Davis and Anderson, 2010). The family is thought to have originated in South America (Anderson, 1990, 2013; Davis et al., 2002b, 2004), an hypothesis that has been corroborated more recently with greatly expanded phylogenetic sampling (Cai et al., unpublished results). The clade has received broad phylogenetic and biogeographic attention (Cameron et al., 2001; Davis et al., 2001, 2002a,b, 2004; Davis and Anderson, 2010), including more focused investigations in the Old World, especially in Africa and Madagascar (Davis et al., 2002a). Efforts to determine finer scale patterns of Malpighiaceae biogeography in the New World, however, have been hampered by taxonomic sampling deficiencies. Along these lines, one area that is ripe for exploration is the Mexican Malpighiaceae flora, which include ~162 species (Anderson et al., 2006 onwards; Anderson, 2013). Malpighiaceae are especially abundant in Mexico's SDTF (Gentry, 1995), where they are represented by ~60 species, putting them in the top five most diverse families in SDTF (Lott and Atkinson, 2006). Based on taxonomic and phylogenetic grounds, Mexican Malpighiaceae have been hypothesized by Anderson (2013) to represent as many as 42 independent origins from outside of Mexico.

The first goal of our study is to test Anderson's hypothesis on the origin of Mexican Malpighiaceae by greatly expanding current taxon sampling for the family (Davis and Anderson, 2010). This will not only facilitate a rigorous assessment of the number of introductions to Mexico (including those inhabitants of SDTF but more broadly in this geographical region), but will also establish the timing and ancestral origins of the diverse Mexican Malpighiaceae flora. This will enable us to distinguish between more ancient long-distance dispersal events directly from South America versus more recent shorter-distance, “stepping stone” dispersal via Central America. This is especially relevant to understanding when Central America became a major corridor for plant migration (Cody et al., 2010; Gutiérrez-García and Vázquez-Domínguez, 2013; Leigh et al., 2014). A related second goal is to understand how rates of dispersal compare to rates of *in situ* diversification, and how these processes shaped the species richness vs. their phylogenetic diversity in Mexico. Finally, the third goal is to investigate if lineages were pre-adapted to SDTF, or if they adapted to this novel biome *in situ*, subsequent to their arrival in Mexico. Relatively few studies have investigated the timing of biome formation using phylogenetic proxies (Becerra, 2005; Davis et al., 2005; Arakaki et al., 2011; Couvreur et al., 2011; De-Nova et al., 2012), yet these approaches hold tremendous promise. It is important to keep in mind, however, using present-day categorizations of biomes as static ecological characters to infer their origin does not fully capture their more dynamic formation over geological time. In particular, different aspects of the abiotic parameters that characterize extant biomes may originate at different times and change at different rates. Here, we seek to elucidate the origination of SDTF in Mexico using two key climate parameters that define this biome today, i.e., overall precipitation (total annual precipitation), and precipitation seasonality (precipitation in the driest quarter). Understanding the dynamics of these interactions is likely to shed key insights into the temporal and spatial nature of the timing of the origin of this biome as it is defined today.

## Materials and methods

### Taxon sampling

Our backbone four gene data set (i.e., plastid [pt] *matK*, *ndhF*, *rbcL*, and nuclear [nu] *PHYC*) was published by Davis and Anderson (2010). It includes 338 ingroup accessions of Malpighiaceae representing all 77 currently recognized genera in the family (Anderson et al., 2006 onwards; Davis and Anderson, 2010). Here, we greatly expanded on this taxon sampling, focusing on Neotropical Malpighiaceae, especially from the Caribbean, Central America, and Mexico (see Table S1 in the Supplementary Material). These additional species are represented in numerous diverse genera, including *Bunchosia*, *Gaudichaudia*, *Mascagnia*, *Stigmaphyllon*, and *Tetrapterys*. Our sampling was guided by W. Anderson (pers. comm.) and is largely summarized in Anderson (2013). Members of Centroplacaceae and Elatinaceae have previously been identified as well supported sister clades to Malpighiaceae (Davis and Chase, 2004; Wurdack and Davis, 2009; Xi et al., 2012), and were included in our analyses as outgroups. *Peridiscus lucidus* Benth. (Peridiscaceae) was used for rooting purposes.

### Molecular methods

Total cellular DNAs were prepared following Davis et al. (2002a) or were obtained from other sources (see Acknowledgments). Voucher information is listed in Table S1.

Amplification and sequencing protocols for obtaining *matK* followed Cameron et al. (2001), using their primers 400F, *trnK*-2R, and 842F; *ndhF* followed Davis et al. (2001); *rbcL* followed Cameron et al. (2001); and *PHYC* followed Davis et al. (2002b) with the addition of forward primer int-1F, which produced an ~800 base-pair (bp) amplicon when paired with reverse primer 623r/cdo (Davis and Anderson, 2010).

Double-stranded polymerase chain reaction (PCR) products were primarily gel extracted and purified using the QIAquick Gel Extraction Kit (*Qiagen*, *Valencia*, *California*, *USA*). PCR products were sequenced in both directions using dye terminators and sequencing protocols at the University of Michigan DNA facility (Ann Arbor, Michigan, USA), MWG Biotechnology (High Point, North Carolina, USA), and GENEWIZ, Inc. (Cambridge, Massachusetts, USA). Chromatograms were assembled into contiguous sequences and checked for accuracy using the software program Sequencher v4.7 (Gene Codes Corporation, Ann Arbor, Michigan, USA). All newly generated sequences were submitted to GenBank (see Table S1).

### Phylogenetic analyses

Nucleotide sequences were aligned by eye using MacClade v4.0 (Maddison and Maddison, 2000); the ends of sequences, as well as ambiguous internal regions, were trimmed from each data set to maintain complementary data between accessions.

Maximum likelihood (ML) bootstrap percentage (BP) consensus trees and Bayesian posterior probabilities (PP) from all individual analyses of the four gene partitions revealed no strongly supported incongruent clades (i.e., >80 ML BP/1.0 PP) and were thus analyzed simultaneously using the search strategies described below.

The optimal model of molecular evolution for the individual and combined analyses was determined by the Akaike Information Criterion (AIC) using ModelTest v3.7 (Posada, 2008). The optimal model was the General Time Reversible model, with rate heterogeneity modeled by assuming that some sites are invariable and that the rate of evolution at other sites is modeled using a discrete approximation to a gamma distribution (GTR+I+Γ). ML analyses of the individual and combined matrices were implemented in the parallelized version of RAxML v7.2.8 (Stamatakis, 2006) using the default parameters. ML BPs were estimated from 100 bootstrap replicates. The Bayesian analyses were implemented with the parallel version of BayesPhylogenies v2.0 (Pagel and Meade, 2004) using a reversible-jump implementation of the mixture model as described by Venditti and Pagel, 2008. This approach allows the fitting of multiple models of sequence evolution to each character in an alignment without *a priori* partitioning. Two independent Markov chain Monte Carlo (MCMC) analyses were performed, and the consistency of stationary-phase likelihood values and estimated parameter values was determined using Tracer v1.5. We ran each MCMC analysis for 10 million generations, sampling trees and parameters every 1000 generations. Bayesian PPs were determined by building a 50% majority-rule consensus tree from two MCMC analyses after discarding the 20% burn-in generations.

### Phylogenetic and divergence time estimation

We used Bayesian methods as implemented in BEAST v1.6.1 (Drummond et al., 2006) to simultaneously estimate the phylogeny and divergence times of Malpighiaceae. A likelihood ratio test rejected a strict clock for the entire dataset (*P*-value < 0.001) and we therefore chose the uncorrelated-rates relaxed clock model, which allows for clade-specific rate heterogeneity.

Our four gene regions were analyzed simultaneously as a single partition using the GTR+I+Γ model as determined using the model selection method described above. Three fossil calibration points served as minimum age constraints for Malpighiaceae and were fit to a lognormal distribution in our analyses. The phylogenetic placement of these calibration points are described in more detail elsewhere (Davis et al., 2002a,b, 2004, 2014). A fossil species of *Tetrapterys* from the early Oligocene (33 Myr, Hably and Manchester, 2000) of Hungary and Slovenia provides a reliable age estimate for the stem node of the two *Tetrapterys* clades. *Eoglandulosa warmanensis* from the Eocene Upper Claiborne formation of northwestern Tennessee (43 Myr, Potter and Dilcher, 1980; Taylor and Crepet, 1987) provides a reliable stem node age for *Brysonima*. Finally, *Perisyncolporites pokornyi* is found pantropically and provides a reliable stem node age for the stigmaphylloid clade (49 Myr, Germeraad et al., 1968; Lowrie, 1982; Berggren et al., 1995; Davis et al., 2001; Jaramillo and Dilcher, 2001; Jaramillo, 2002). The root node, which we set to a normal distribution of 125 ± 10 Myr, corresponds to the approximate age of the eudicot clade. This represents the earliest known occurrence of tricolpate pollen, a synapomorphy that marks the eudicot clade, of which Malpighiales are a member (Magallón et al., 1999; Stevens, 2001 onwards).

MCMC chains were run for 10 million generations, sampling every 1000 generations. Of the 10,001 posterior trees, we excluded the first 2000 as burn-in. Convergence was assessed using Tracer v1.5 (Rambaut and Drummond, 2007).

### Distribution and climate data

Malpighiaceae are distributed widely throughout the Old and New World tropics. We characterized the geographic ranges for each species into seven regions abbreviated as follows (Table S2, Figure S1): SA, South America; CA, Central America; Me, Mexico; Ca, Caribbean; As, Asia; Af, Africa; and M, Madagascar. Widespread species were assigned to more than one area. The maximum observed geographic range included four regions (SA, CA, Me, and Ca). The species' occurrence within these regions was based on Anderson et al. (2006 onwards; pers. comm.).

In addition to this broad geographic classification, we also obtained geo-referenced occurrence data from the Global Biodiversity Information Facility database (www.gbif.org) on May 07, 2012. These data, which included 28,254 initial records, were subsequently filtered based on several criteria. They were scrubbed to exclude species that were not included in our phylogeny, and of occurrences that fell outside of a species' geographic distribution according to Anderson et al. (2006 onwards). This helped to eliminate misidentified specimens or specimens with incorrect locality information. After filtering, our database included 14,743 geo-referenced data points representing 357 species (median number of records for each species = 8). Plant synonymy was checked against the University of Michigan Herbarium Malpighiaceae website (http://herbarium.lsa.umich.edu/malpigh/). We developed a python web scraping script to automate this procedure (https://github.com/Bouteloua/MalpighiaceaeTaxaScraper).

Climate data for total annual precipitation (mm, bio12) and precipitation of the driest quarter (mm, bio17) was extracted from the World Bioclim dataset at 30″ resolution (www.worldclim.org) based on geo-referenced data and averaged for each species.

### Ancestral climate and biome estimation

To estimate ancestral climate states for total annual precipitation and precipitation during the driest quarter, we used a standard Brownian motion model with parameters fitted with ML and Bayesian MCMC. These methods were implemented in the R package *phytools* v0.3–93 (Revell, 2012) using the functions “anc.ML” and “anc.Bayes,” respectively. Bayesian models were run for 10,000 generations. We estimated ancestral climate states for each of the 100 ML bootstrap trees.

### Evolutionary lag time

Adaptation to a new habitat can occur after, before, or during a geographic migration event. In the event that adaptation occurs after a lineage has migrated to a new region, it is known as an “evolutionary lag.” To investigate whether there was an evolutionary lag in the adaptation to dry forest habitats after lineages became restricted to Mexico, we calculated the difference between the age of each geographic restriction to Mexico and the age that a lineage first evolved either a given minimum climatic threshold.

**Figure 2:**
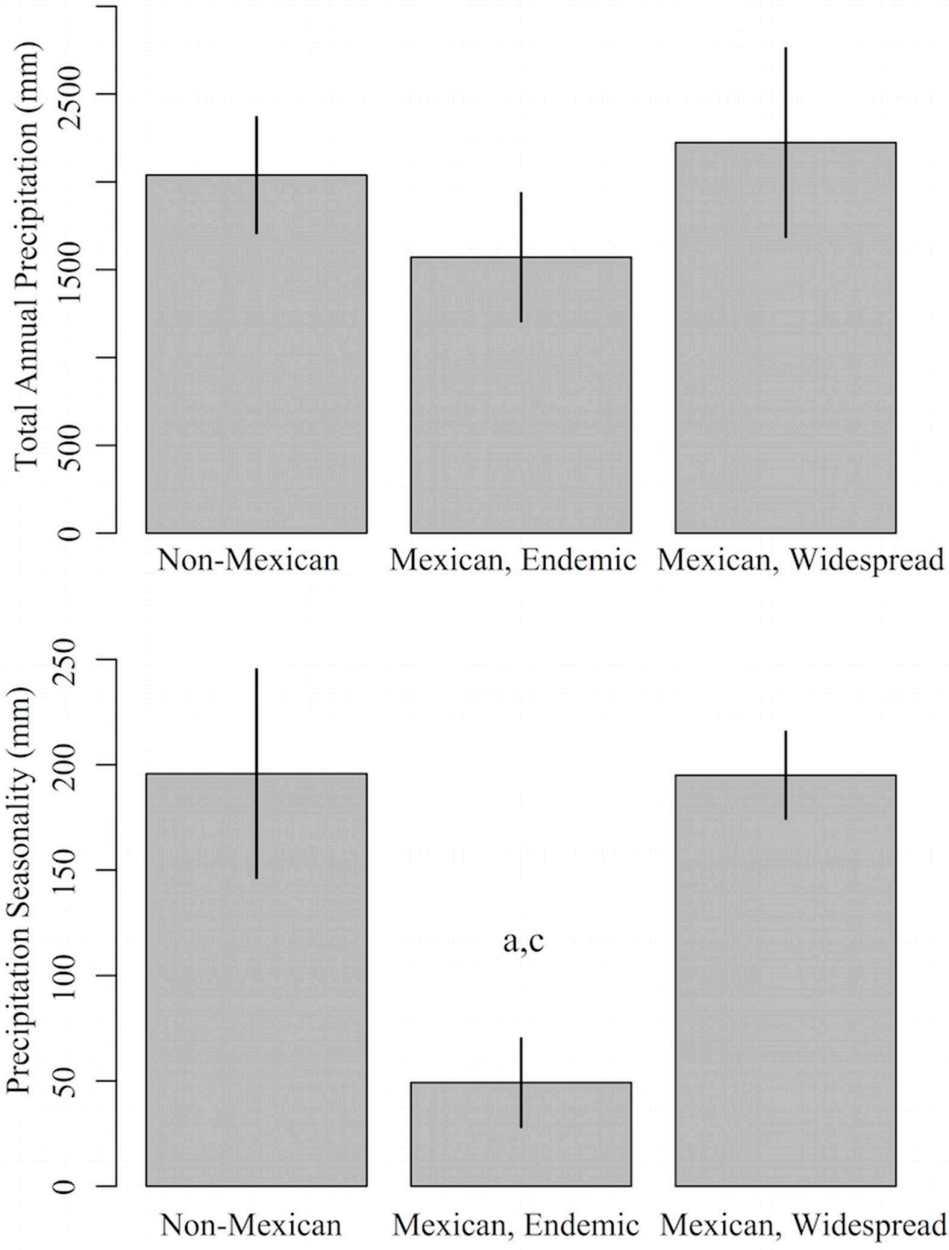
**Precipitation profiles of Mexican and non-Mexican Malpighiaceae**. Mexican Malpighiaceae are subdivided into two groups: endemic lineages that are geographically restricted to Mexico, and wide-ranging lineages that also occur outside of Mexico. The two climate variables are: total annual precipitation (mm yr^−1^) and seasonal precipitation (total precipitation during the driest quarter, mm yr^−1^). Error bars indicate standard errors. Significant (*P* < 0.05) differences in mean values between groups based on a comparison with phylogenetic generalized linear models are indicated by a (Non-Mexican), b (Mexican, Endemic), and c (Mexican, Widespread).

Minimum climatic values were based on current definitions of SDTF (Murphy and Lugo, 1986; Pennington et al., 2009). SDTF is defined by total annual precipitation of ≤ 1800 mm and a seasonally dry period of 6 months with ≤ 100 mm of precipitation. For total annual precipitation, we estimated the lag time for two minimum thresholds: 1800 mm yr^−1^ (in line with the traditional definition of the biome) and 1600 mm yr^−1^ (in line with the observed value for endemic Mexican Malpighiaceae, see Figure 2, Table S8). For the seasonally dry period, we estimated precipitation during the driest quarter (3 months) for two minimum thresholds: 50 mm qtr^−1^ (in line with the traditional definition of the biome) and 100 mm qtr^−1^ (in line with a less severe seasonality).

### Climatic profile of mexican malpighiaceae

To characterize the climate profiles of Malpighiaceae, we used phylogenetic generalized linear models (PGLM; Revell, 2010) to test how Mexican Malpighiaceae differed from other Malpighiaceae with regard to total annual precipitation and precipitation during the driest quarter. Models were analyzed in R v3.0.2 (R Team Core, 2013) using the “pgls” function implemented in *caper* v0.5 (Orme et al., 2013). We compared two groups of Mexican Malpighiaceae with non-Mexican Malpighiaceae at large. The first group of Mexican Malpighiaceae included species that were geographically restricted (i.e., endemic) to Mexico. Species in the second group were more widespread, occurring in Mexico, as well as, in regions outside of Mexico. These analyzes were run across all 100 ML bootstrap trees.

### Biogeographic range reconstruction

Reconstructing the biogeographic history of Malpighiaceae was estimated using the dispersal-extinction-cladogenesis model (DEC; Ree and Smith, 2008) modified to incorporate founder-event speciation (DEC+J; Matzke, 2013). The DEC+J model assumes dispersal-mediated range expansion, extinction-mediated range contraction, and founder-event speciation with the probability of either event occurring along a particular branch being proportional to the length of that branch and the instantaneous transition rates between geographic areas (Ree and Smith, 2008; Matzke, 2013). Of the 480 species included in our original phylogenetic inference and divergence time estimation, 395 had enough available distribution data to be included in the biogeographic analysis.

We initially compared the DEC+J model with the standard DEC model, as well as the DIVA and BAYESAREA models to determine their fit to our data. Model-fit was assessed by comparing weighted AIC scores (Matzke, 2013). The DEC+J model was the best fit to our data, and was subsequently used for all following analyses (Table S7).

We used the R package *BioGeoBEARS* v0.2.1 (Matzke, 2013) to obtain the most likely dispersal scenarios at all internal nodes of 100 ML bootstrap trees (with outgroups removed) under the DEC+J model.

In our biogeographic model, we restricted the number of regions a lineage can inhabit to the maximum number of regions observed among extant taxa (four of seven). We altered the migration probabilities among geographic areas to reflect changes in connections over geological time (Mao et al., 2012). These migration probabilities range from 0.1 for well-separated areas, to 1.0 for contiguous landmasses. We devised separate migration matrices for four discrete time intervals: 70–45 Myr, 45–30 Myr, 30–5 Myr, and 5–0 Myr. The use of non-zero migration probabilities allowed for the possibility that lineages could have a range that includes regions that are separated today but were once connected. They also allowed for changes in the probability of dispersal between regions that were once separated by large distances before becoming nearly contiguous.

To calculate “dispersal rate” into Mexico, we calculated the average number of expansions into Mexico per million years. Furthermore, we tested for changes in dispersal rate, by estimating an inflection point across the age of expansions into Mexico. To test for an inflection point, we used the function “findiplist” in the R package *inflection* v1.1 (Christopoulos, 2012). We subsequently re-calculated the migration rates for expansions on either side of the estimated inflection point.

### Taxon sampling biases

Biases in taxon sampling can introduce errors in estimates of geographic range and ancestral biome. To address potential taxon sampling biases across geography and biomes we utilized our GBIF records as a reference. This dataset includes 828 species of Malpighiaceae (357 of which we sampled in our phylogeny). While this GBIF dataset does not include all of the species in the family, it provides a very broad representation across all sampled biomes and geographic regions relevant to our analyses. Thus, it can be used to assess biome and geographical biases in the taxa included in our phylogeny.

For each genus, we compared expected species counts per biome/geographic region based on GBIF against species counts in our phylogeny using a standard χ^2^-test. The affinity of each species biome was scored based on the majority occurrence of the species in the World Wild Fund terrestrial biome map (http://www.worldwildlife.org/science/wildfinder/). Of the 72 genera sampled in our study, eight (*Acridocarpus*, *Banisteriopsis*, *Bunchosia*, *Byrsonima*, *Diplopterys*, *Heteropterys*, *Hiraea*, and *Tetrapterys*) exhibited biases in biome sampling (Table S3), while only four (*Byrsonima*, *Heteropterys*, *Hiraea*, and *Stigmaphyllon*) exhibited biases in geographical sampling across the New World (Table S4). The biases were primarily limited to our sampling of Central American SDTF taxa (Table S5). The removal of these genera, however, did not affect our general conclusions regarding the lag time for the adaptation of lineages to seasonally dry tropical forests (Table S6).

### Diversification rates

We tested for shifts in net species diversification (speciation—extinction) rate through time and among lineages using BAMM v1.0 (Rabosky, 2014). BAMM allows for simultaneous estimates of rate shifts across a phylogeny using a Bayesian framework. BAMM can also account for incomplete taxon sampling by lineage. We included estimates of lineage completeness by taking the proportion of species within a given genus present in our phylogeny relative to the total number of species reported to be in the genus (Table S9). Additional priors were set using the “setBAMMpriors” function in the R package *BAMMtools* v1.0.1 (Rabosky et al., 2014). For each of 100 ML bootstrap trees, we conducted one run with 10 million generations of MCMC sampled per run, sampling parameters every 10,000 generations. We discarded the first 10% of each run as burn-in. We computed effective sample size (ESS) for log-likelihood and rate parameters to assess the convergence of each run. All parameters had effective sample sizes >400. We calculated the mean of the marginal posterior density of the net diversification rate for small segments (τ = 0.5) along each branch for every tree using the *dtRates* function. This allowed us to assess the variation in diversification rates across every tree.

## Results

### Phylogenetic inference and divergence time estimation of malpighiaceae

Our taxon sampling includes 461 accessions (413 taxa identified to species and 28 taxa identified to genus) representing ~35% of the total species diversity of Malpighaiceae. In addition, we included 19 taxa from across the Malpighiales as outgroups. The aligned pt *matK*, *ndhF*, *rbcL*, and nu *PHYC* data sets included 1194, 867, 1414, and 1180 base pairs (bp), respectively. The data matrix presented in this study is available in TreeBase (ID# 16087).

Our phylogeny is congruent with previous results (Cameron et al., 2001; Davis et al., 2001; Davis, 2002; Davis and Anderson, 2010), but represent an improvement over previous studies. Our increased sampling here is particularly relevant because it greatly enhances our ability to investigate fine scale diversification patterns in the Neotropics, especially related to Mexican Malpighiacae. Our sampling included 95 species from Mexico, 47 of which are endemic to the region. In addition, several new taxa from adjacent areas of Central America, the Caribbean, and South America were also sampled. Well-supported relationships were congruent between analyses of individual data sets, and the data were thus analyzed in combination. For the sake of space, we present the ML results of the combined four-gene analysis here (see Figure S2). Similarly, our divergence time estimates are congruent with previous estimates (Davis et al., 2002b, 2004, 2014).

### Climatic profile of sampled mexican malpighiaceae

Our sampled Malpighiaceae that are geographically restricted to Mexico tended to have lower total annual precipitation, when compared to Malpighiaceae that are not endemic to Mexico, but this difference was not significant (Table S7, Figure 2). However, Malpighiaceae restricted to Mexico did differ significantly with regard to precipitation seasonality (Table S8, Figure 2). Namely, Malpighiaceae restricted to Mexico had, on average, a significantly distinct dry season with an average precipitation of ~49.2 mm during the driest quarter. This is compared to an average precipitation during the driest quarter of 195.8 mm for non-Mexican Malpighiaceae, and 195.0 mm for Malpighiaceae that occur in, but are not restricted to Mexico.

### Biogeographic range reconstruction

**Figure 3:**
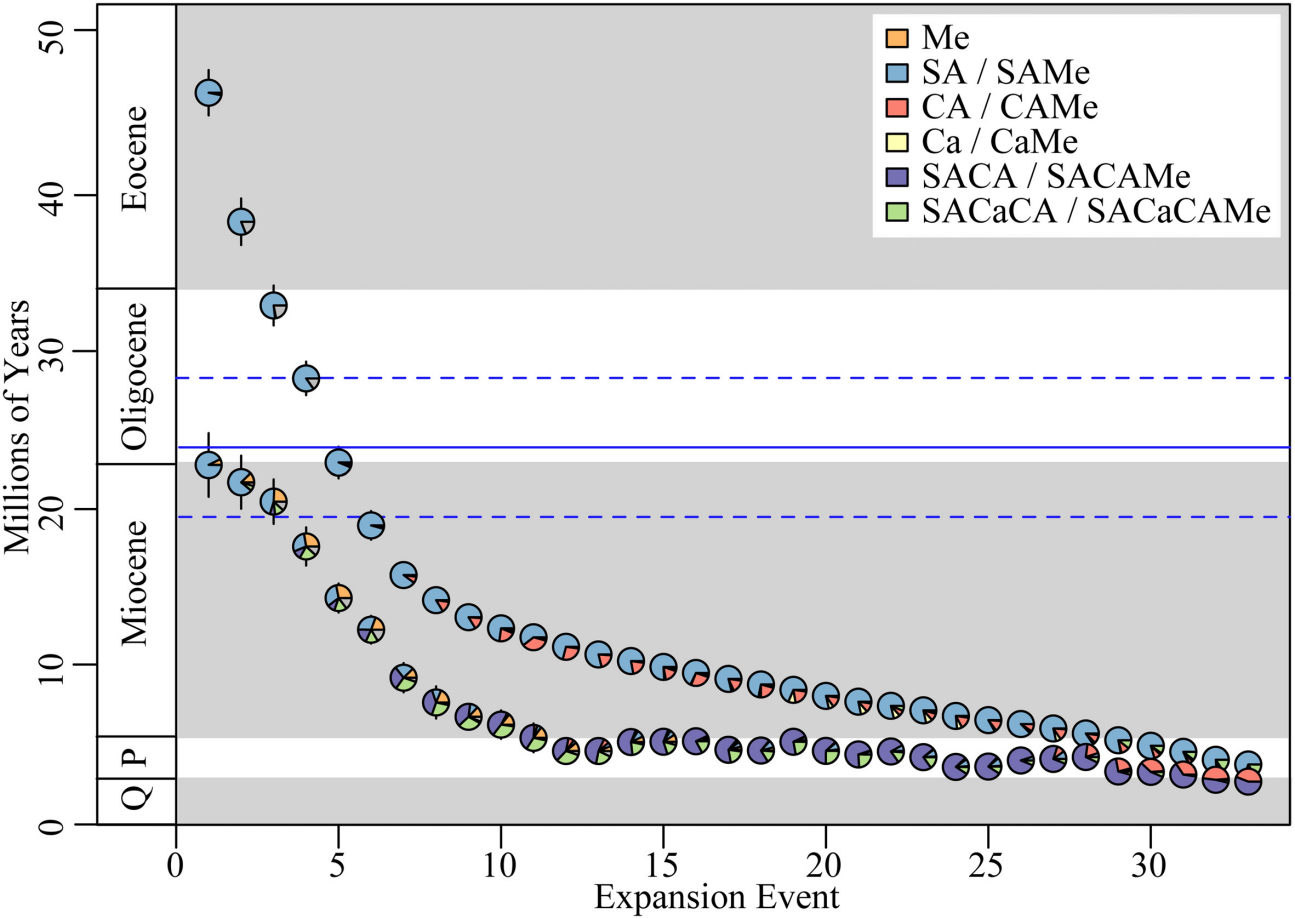
**Biogeographic expansions into Mexico**. Mean date of each expansion event estimated from Lagrange for both stem, parent nodes (upper row) and crown, daughter nodes (lower row). Error bars represent 95% confidence intervals estimated across mean ages from 100 ML trees. Plot ordered by age of stem node from oldest to youngest. Pie-charts represent the estimated proportion of the ancestral range at each respective node. Parent node range represents the original range, prior to dispersal into Mexico, while the daughter node range represents the range post-dispersal, which includes Mexico. Key indicates ancestral range reconstructions for stem, parent nodes and daughter, crown nodes to left and right of backslash, respectively. Horizontal bold, blue line indicates the inferred inflection point (solid = mean, dashed = 95% quantile range) when the rate of dispersal between North and South America increases significantly (see Figure 5). *P*, Pliocene; Q, Quaternary (Pleistocene/Holocene).

**Figure 4:**
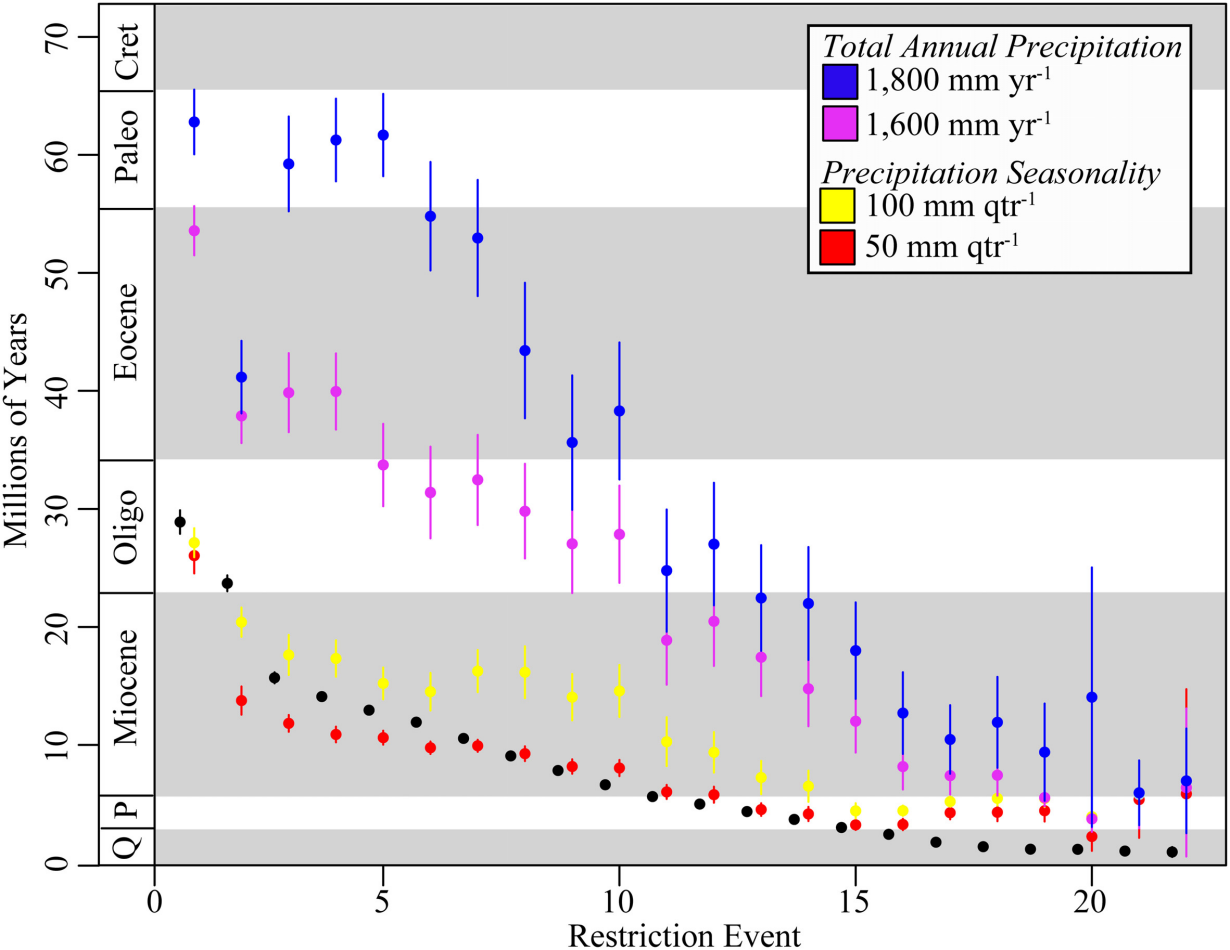
**Age of geographic restriction to Mexico, and adaptations to seasonal dry tropical forest climate in Mexico**. Mean age of geographic restrictions to Mexico shown with black circles. Mean age of the evolution of total annual precipitation (≤1800 mm yr^−1^; blue circles) and seasonal precipitation (≤50 mm qtr^−1^; red circles) in Mexican endemics as defined for modern seasonally dry tropical forest shown with blue and red circles, respectively. Also included is (i) the mean age of the evolution of total annual precipitation under more extreme conditions exhibited by extant Malpighiaceae in Mexico (≤1600 mm yr^−1^; light blue) and (ii) seasonal precipitation under more moderate historic conditions (≤100 mm qtr^−1^; yellow). Error bars represent 95% confidence intervals estimated across mean ages from 100 ML bootstrap trees. Plot ordered by age of restriction event from oldest to youngest. Cret, Cretaceous; Paleo, Paleocene; P, Pliocene; Q, Quaternary (Pleistocene/Holocene).

We identified an average of ~33 (Quantile_95%_: 29–38) independent range expansions into Mexico, i.e., a transition where the ancestral range of a lineage expands to include Mexico, but is not necessarily restricted to Mexico (Figure 3). These expansions were inferred to have occurred at the stem group node, and originate predominantly from South America. Furthermore, they often include the colonization of additional regions, most commonly Central America, and to a lesser extent the Caribbean. We also identified an average of ~22 (Q_95%_: 19–26) range restrictions to Mexico i.e., a transition where the ancestral range of a lineage becomes restricted to Mexico (Figure 4). The majority of these restrictions involved lineages where the ancestral range include both Mexico and other additional regions, with the restriction to Mexico occurring after the lineage went extinct in the ancestral non-Mexican regions (Figure S3).

**Figure 5:**
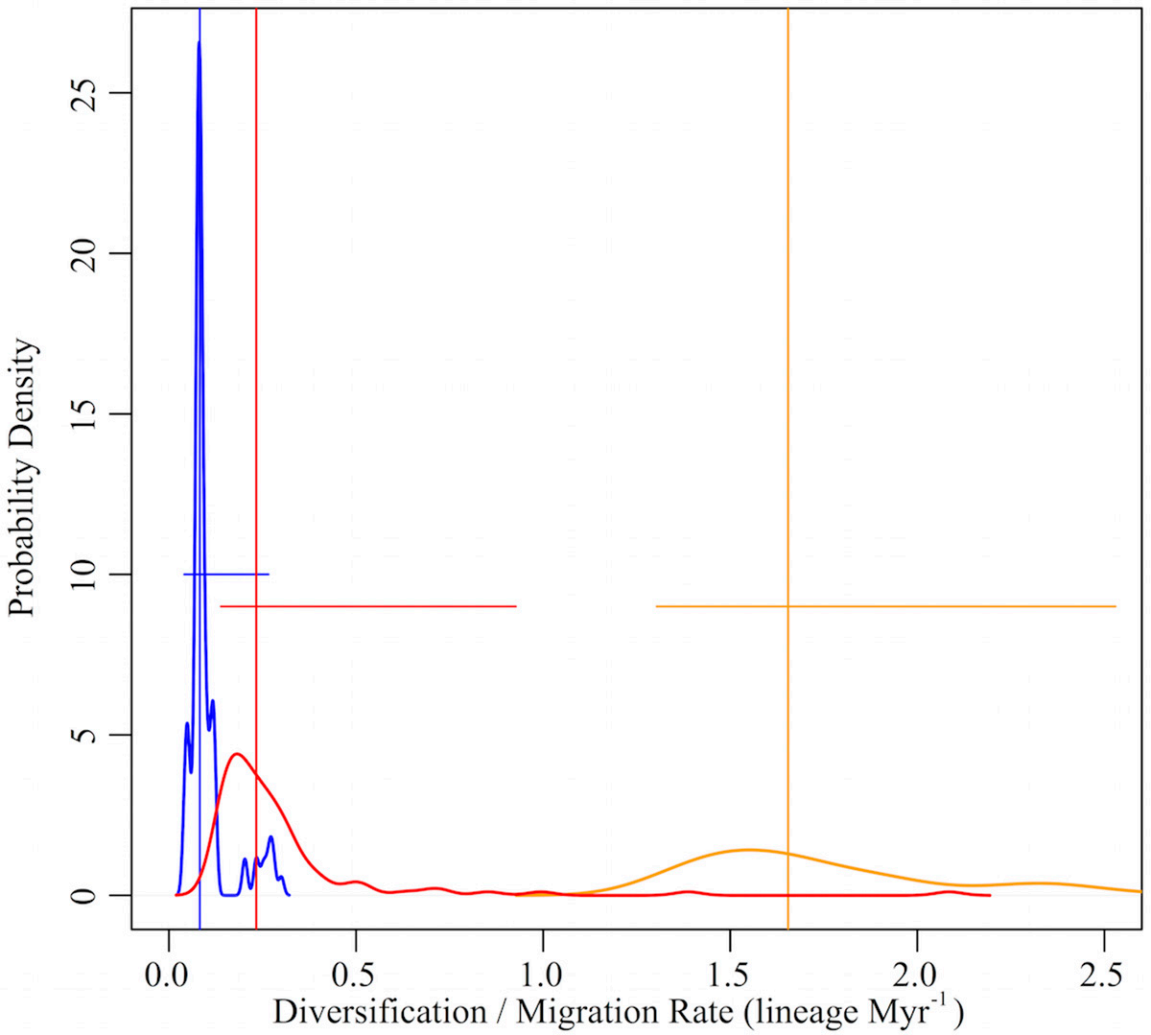
**Dispersal vs. diversification rates of Mexican Malpighiaceae**. Dispersal rates represent the number of lineages migrating into Mexico, predominately from South America, per million years. The dispersal rate is divided into two categories that reflect a significant change in the rate of migration between North and South America corresponding with the hypothesized development of Central American corridors in the late Oligocene and early Miocene (i.e., dispersal rate inflection point, Figure 3). The dispersal rate before the inflection point is shown in red, while the dispersal rate after the inflection point is shown in orange, respectively. Diversification rates are based on estimates of speciation and extinction across all Malpighiaceae to illustrate the full range of diversification rates across the entire family (blue). Vertical lines indicate median rate values; horizontal lines indicate the 95% quantile range for each rate. Both dispersal and diversification rates were estimate across 100 ML bootstrap trees.

The mean migration rate (based on expansion events, as defined above) of lineages into Mexico was 0.8 lineages Myr^−1^ (Q_95%_: 0.6–1.2 lineages Myr^−1^). The migration rate was not uniform through time, however. We identified an inflection point in the migration rate at 23.9 Myr (Q_95%_: 19.5–28.3 Myr; Figure 5). The migration rate prior to the inflection point was 0.3 lineages Myr^−1^ (quantile_95%_: 0.1–0.9 lineages Myr^−1^), while the rate after the inflection point increased significantly to 1.7 lineages Myr^−1^ (Q_95%_: 1.3–2.5 lineages Myr^−1^).

### Pre-adaption vs. *in situ* adaptation post colonization

The evolution of total annual precipitation characteristic of SDTF occurred well before the evolution of either geographic restrictions to Mexico (Figures 4, S4). For total annual precipitation ≤ 1800 mm yr^−1^, the mean lag time was 22.0 Myr before the geographic restriction to Mexico, with values ranging from 39.4 Myr before to 30.0 Myr after the restriction to Mexico (Q_95%_: 36.0–21.6 Myr) (Figure S4). At an even stricter threshold consistent with modern SDTF, total annual precipitation ≤ 1600 mm yr^−1^, the lag time was 15.8 Myr before the geographic restriction to Mexico, with values ranging from 43.8 before to 2.2 Myr after a restriction to Mexico (Q_95%_: 23.3–11.6 Myr) (Figures 4, S4).

The evolutionary lag of precipitation seasonality characteristic of SDTF was dependent on the age of the geographic restriction to Mexico (Figures 4, S5). For precipitation seasonality ≤ 50 mm qtr^−1^, there were, on average, four lineages that adapted to seasonality before a restriction to Mexico, 10 lineages that adapted to seasonality after a restriction to Mexico, and six lineages that adapted to seasonality concurrent with a restriction to Mexico (Figures 4, S5). Lineages that adapted to seasonality after becoming restricted to Mexico tended to be older, while younger lineages tended to be pre-adapted to seasonally dry periods (Figures 4, S5).

### Diversification rates

There were, on average, four major shifts in diversification rate across the Malpighiaceae. These shifts were primarily driven by increases in speciation rate (Figure S6). Two of these shifts were associated with lineages that include high species diversity in Mexico, *Galphimia* (22 sp.) and *Bunchosia* (20 sp.). The additional shifts in diversification rate were at the base of the *Byrisonima* clade, and earlier in the Malpighiaceae (at the most recent common ancestor of the hiraeoid and tetrapteroid clades sensu Davis and Anderson, 2010). The mean diversification rate for Malpighiaceae was 0.08 lineages Myr^−1^ (Q_95%_: 0.04–0.27 lineages Myr^−1^).

## Discussion

We have structured our discussion below to focus first on the arrival of Malpighiaceae into Mexico as a geographic entity. Here, we detail the broader biogeographic context of these immigrants, including their ancestral areas of origin and timing of arrival. For the second part of our discussion we explore the relative contribution of *in situ* lineage diversification vs. dispersal-mediated processes in forming the Malpighiaceae species pool in Mexico, which gave rise to SDTF there. And third, we focus on the numerous immigrants to Mexico that have become geographically restricted (i.e., endemic) to this region. Mexican endemic Malpighiaceae are overwhelmingly represented in the SDTF (Figure S7), and thus represent a key to understanding the aridification of Mexico and the formation of this important biome. Here, we focus on characterizing the temporal and spatial nature of the origin of SDTF by investigating if these adaptations arose before or after these lineages became endemic to Mexico.

*Mexican Malpighiaceae represent numerous dispersal events from South America*. Our analyses indicate that the ~162 species that constitute the Mexican Malpighiaceae flora represent ~33 independent introductions from outside Mexico (Figure 3). These estimates corroborate the large number of introductions to Mexico hypothesized by Anderson (2013) based on his knowledge of the phylogeny and taxonomy of the family. Our ancestral area reconstructions further identify South America as the ultimate source of these many Mexican clades, which is supported by earlier evidence that the origin and early diversification of the family occurred in South America (Anderson, 1990; Davis et al., 2002b, 2004). These findings more generally corroborate the striking floristic similarities between dry-forested regions in northern South America and Mexico (Linares-Palomino et al., 2011).

The migration of South American Malpighiaceae into Mexico falls into two categories. The first involves more ancient northward migrations of South American ancestors directly into Mexico (Figure 3). These six migrations began during the Eocene and continued into the early Miocene (46–19 Myr): the first three occur between the Eocene–mid Oligocene (46–33 Myr) and involve disjunct distributions between South America and Mexico; the remaining three occur between the mid-Oligocene–early Miocene (28–19 Myr) and involve disjunct distributions between South America and Mexico or occupancy of Mexico alone. Based on our ancestral range reconstructions, these migrations do not appear to be facilitated by intervening connections involving Central America or the Caribbean. Taxon sampling bias, while a concern for some lineages, is not a likely explanation for the general pattern we observe (see Materials and Methods). Given the physical distance between these two regions for at least the earliest three dispersal events, long-distance dispersal is likely the predominant mode of migration from South America to Mexico. The three more recent dispersals occur during a time period when we believe Central American corridors were becoming at least partially available for migration, which perhaps explains the increased ambiguity in these ancestral range reconstructions (Figure 3).

The second category of migration from South America to Mexico includes the majority of events, and implicates a “stepping-stone” dispersal scenario, with Central America, and to a lesser extant the Caribbean, serving as a bridge between South America and Mexico (Figure 3). Here, a remarkable 17 of the 33 Mexican Malpighiaceae clades resulting from northward migrations originating in South America had ancestral ranges that expanded to also include Central America. These migratory events begin in the early Miocene ~16 Myr, and continued until as recently as the early Pleistocene, ~2 Myr. Collectively, these findings corroborate a wide range of studies indicating that dispersal plays a major role in tropical forest assembly (Dick et al., 2003; Lavin et al., 2004; Clayton et al., 2009).

A broader geographical framework that more precisely incorporates the timing of these dispersal events into Mexico provides a more nuanced context for interpreting the biogeographic origins of the Mexican Malpighiaceae flora. South American migrations to Mexico occur with striking regularity over an extended period beginning in the Eocene and continuing to the Pliocene (46.4–3.8 Myr). During this period we observe an average rate of 0.8 migrations Myr^−1^. This average, however, obscures a far more dynamic pattern in the change in the rate of migration. At ~23.9 Myr (Q_95%_: 19.5–28.3 Myr) we infer a significant, six-fold increase in the rate of migration between North and South America.

This early Miocene shift in the pattern and rate of migration likely sheds key insights into geological processes that shaped Neotropical biogeography. The Cenozoic paleoland and paleoclimatic reconstructions for Central America, especially involving the Central American Seaway and the rise of the Isthmus of Panama, are contentious (Klocker, 2005; Molnar, 2008). Although numerous lines of geological and biological evidence support a more recent Pliocene shoaling of the Isthmus (Molnar, 2008; Leigh et al., 2014), which established the first direct connection between North and South America, evidence from geology and molecular divergence time estimates in various clades indicate that this barrier was partly permeable to plant migration beginning at least by the Oligocene (Montes et al., 2012; Gutiérrez-García and Vázquez-Domínguez, 2013; Leigh et al., 2014). Our results demonstrate a combination of early long-distance dispersal between South America and Mexico in the Eocene–Oligocene, followed by a shift in the early Miocene, when we see a dramatic increase in the rate of migration, likely due to an increased potential for shorter-distance dispersal. These results suggest that sufficient Central American land corridors were available for plant migration between North and South America, well in advance of when the geological connection between these continents appears to have been fully established ~3.0 Myr (Leigh et al., 2014). Additionally, our analyses of xeric adaption discussed below indicate that these Central American corridors would likely have been characterized by drier forest biomes, in particular, forests with relatively low annual precipitation.

Finally, fruit dispersal syndromes summarized by Anderson (2013) provide ancillary support for these two hypothesized dispersal routes to Mexico–i.e., direct long-distance dispersal to Mexico from South America vs. “stepping-stone” dispersal via Central America. The two clades implicated in older long-distance dispersal events to Mexico from South America, *Bunchosia* and *Byrsonima*, are characterized by fleshy bird-dispersed fruits. While the influence of dispersal morphology on the potential for long-distance dispersal remains controversial (Higgins et al., 2003; Nathan, 2006), our results provide ancillary evidence indicating that bird dispersed lineages were more likely to disperse long-distances without an intervening land connection such as Central America. This is corroborated by other empirical studies that have found an increased propensity for long-distance dispersal among bird dispersed plants in the tropics (Hardesty et al., 2006; Jordano et al., 2007). In contrast, the vast majority of Malpighiaceae that successfully made the more recent migrations possess wind-dispersed fruits, including species with winged samaras (*Adelphia*, *Bronwenia*, *Banisteriopsis*, *Calcicola*, *Callaeum*, *Carolus*, *Christianella*, *Cottsia*, *Diplopterys*, *Gaudichaudia*, *Heteropterys*, *Hiraea*, *Mascagnia*, *Psychopterys*, *Stigmaphyllon*, *Tetrapterys*) and bristly fruits (*Echinopterys*, *Lasiocarpus*). These fruits, while not ideal for traversing large bodies of open ocean, were likely sufficient for making shorter step-wise dispersals to Mexico when facilitated by intervening land masses with the rise of Central America. *Malpighia*, which possesses mostly bird-dispersed fleshy fruits, is an exception in this regard. They are one of the more recent inhabitants of Mexico (expanding into Mexico ~5 Myr) that migrated to that region via Central America. On the basis of fruit morphology, however, Anderson (2013) hypothesized that *Malpighia* may have colonized Mexico via winged, wind-dispersed ancestors, which is consistent with our broader interpretation of dispersal patterns. A final conundrum is *Galphimia*, which represents the oldest introduction to Mexico. Although the petals of *Galphimia* species in Mexico are persistent and thus may facilitate wind-dispersal, their fruits otherwise break apart into dry cocci that exhibit no obvious adaptation for long-distance dispersal (Anderson, 2013).

Dispersal mediated processes dominate initial species pool formation and contribute disproportionately to the phylogenetic diversity of Mexican Malpighiaceae. Our analyses of species diversification rates illuminate the formation of the initial species pool of Malpighiaceae that gave rise to SDTF in Mexico. The vast majority of the ~33 South American lineages that dispersed into Mexico exhibit no evidence of accelerated net diversification rates (Figure S6). Beginning in the Miocene, when rates of migration increased significantly in conjunction with the development of land corridors through Central America, the rate of Malpighiaceae dispersal between South America and Mexico was 1.7 lineages Myr^−1^, outpacing even the highest rate of net species diversification, 0.3 lineage Myr^−1^ (Figures 3, 5). This nearly six-fold difference in the rate of dispersal vs. diversification establishes the former as key factor in the initial establishment of the Mexican Malpighiaceae species pool. The lone exceptions to this pattern of relatively low net diversification are the two oldest introductions to Mexico in the Eocene and Oligocene, *Galphimia and Bunchosia*. These two clades exhibit significantly increased net diversification rates. Although we cannot pinpoint the precise geographic location where these two clades began to diversify, their average diversification rate is on par with the rate of dispersal we observe into Mexico following the development of a Central American migratory corridor.

Our results collectively highlight the balance between *in situ* diversification vs. dispersal in forming a regional species pool, and how this balance can change with time. Here, the two lineages that demonstrate prolific diversification in the family, *Galphimia* (22 sp. in Mexico; Anderson, 2013) and *Bunchosia* (20 sp. in Mexico), constitute roughly 24–28% of the ~162 extant Mexican Malpighiaceae. Beyond these exceptional clades, however, it is remains clear that even the large number of independent dispersal events into Mexico (~33) that formed the initial species pool cannot account entirely for the extant diversity of Mexican Malpighiaceae (~162 sp). Thus, *in situ* diversification is clearly an important contributor to the extant species richness of this region. At the same time, however, our results indicate that the predominant source of increased phylogenetic diversity (PD) in Mexican Malpighiaceae is attributed largely to dispersal. Using the stem group age of each introduction to Mexico as a conservative assessment of PD generated via *in situ* diversification (as measured by mean phylogenetic distance; Webb et al., 2002), we found that the mean phylogenetic distance (i.e., the mean branch length between any pair of taxa) between relatives that arose via *in situ* diversification was 12.5 Myr (Q_95%_ = 10.4-14.9 Myr). In contrast, mean phylogenetic distance between the Mexican subclades that arose via dispersal into the region was substantially larger, 100.5 Myr (Q_95%_ = 96.4-104.6 Myr). It is clear from these results that dispersal has contributed substantially to PD in Mexico by effectively “sampling” broadly from the entire Malpighiaceae clade. The contribution of dispersal to PD we identify may be a general pattern facilitating the formation of SDTF in Mexico, and to the formation of regional species pools more broadly. The *Oxalis* of the Atacama Desert, for instance, represent a broad diversity of *Oxalis* clades that independently dispersed into this region (Heibl and Renner, 2012).

What limited the majority of introduced lineages from radiating in Mexico? One hypothesis is that newly established lineages did not exhibit a competitive advantage to more recently introduced lineages. Thus, newly arriving lineages were able to occupy SDTF in Mexico with a frequency commensurate with the apparent richness of their source pool outside of Mexico (especially from South America). Support for this hypothesis comes from the fact that we find the vast majority of immigrant lineages arrived pre-adapted to at least one of the two traits that characterizes SDTF, annual precipitation. However, most lineages were not adapted to seasonally dry periods. A second hypothesis is that SDTF is more generally dispersal-limited, thus restricting the ability of lineages to radiate subsequent to their arrival into expanding habitat. Such a scenario might offer newly available niche space for lineages to occupy via dispersal from outside the regional source pool. Previous studies have found a significant degree of genetic differentiation among geographically isolated populations across dry forest habitats, supporting the idea that these dry-forests are dispersal-limited (Pennington et al., 2006a, 2009). Furthermore, at least one of the two lineages (*Bunchosia* [20 sp.], and less significantly so *Malpighia* [19 sp.]) that diversified substantially in Mexico are bird dispersed. Such lineages are likely to more easily migrate and radiate across a landscape via allopatric speciation. This is compounded by the fact that the formation of SDTF was not likely contiguous, both spatially and temporally (Graham and Dilcher, 1995). New fragments of SDTF likely arose at different times and in different regions of Mexico, which might have greatly facilitated the continuous establishment of new Malpighiaceae lineages via dispersal from external source pools. In combination, these processes might act to limit the overall diversification of single Mexican introductions, and help explain the pattern of frequent and continued dispersal of new lineages into this biome.

The predominance of dispersal in the formation of a regional species pool has been shown elsewhere, including in the California chaparral (Ackerly, 2004), the Atacama Desert (Heibl and Renner, 2012), and in temperate, post-glacial habitats (Williams et al., 2004). However, these and related studies are commonly restricted to a narrow window of time or to a single, relatively young clade. In our case, the overwhelming pattern of dispersal we identify spans a wide period of time as well as a large, diverse plant clade. This raises the possibility that the characterization of species pools being dominated by single lineages that moved into a region and subsequently radiate, such as in the Andes (Hughes and Eastwood, 2006) or on remote islands (Baldwin and Sanderson, 1998; Emerson, 2002), may be exceptions rather than the rule of species pool assembly. A more general pattern, especially with regard to the phylogenetic diversity of a species pool, might instead be the steady recruitment of independent lineages via dispersal over tens of millions of years, most of which exhibit modest rates of *in situ* diversification.

*Endemic Mexican Malpighiaceae exhibit both ex situ* pre-adaptation *and in situ adaption to xeric environments in Mexican SDTF*. Our analyses of trait adaptation to precipitation provide direct insights for more clearly interpreting the spatial and temporal origins of xeric adaptations by Mexican Malpighiaceae. An emerging question in the field of biogeography is when did traits relevant to community assembly originate (Ackerly, 2004; Cavender-Bares et al., 2009; Simon et al., 2009; Edwards and Donoghue, 2013). Specifically, did Mexican Malpighiaceae evolve adaptations to xeric habitats elsewhere (*ex situ*, i.e., were they pre-adapted?) followed by movement into Mexico or did they evolve these adaptations *in situ* in Mexico. This question has been more succinctly phrased as “is it easier to move, than to evolve?” (Donoghue, 2008). To explore this question in Mexican Malpighiaceae, we focused on two key traits central to the definition of contemporary Mexican SDTF: lower total annual precipitation (≤1800 mm) and extreme seasonality in precipitation (≤50 mm over the driest 3 months). Here, we specifically examined the ~22 Mexican lineages that have become endemic to Mexico (Figure 5), since these lineages are overwhelmingly represented in SDTF of this region (Figure S4).

We found that the timing of adaptation to these two key climate traits contrast sharply in relation to when lineages become restricted to Mexico (Figures 4, S4, S5). Adaptation to drier annual precipitation evolved well before Malpighiaceae become endemic to Mexico by an average of ~15 Myr (Q_95%_: -1.3 to 64.8 Myr) (Figures 4, S4). Thus, lineages that subsequently became geographically restricted to Mexico were likely pre-adapted for living in relatively drier environments. This climatic adaptation appears to have arisen largely in South America (Figure S4). In contrast, adaptation to extreme precipitation seasonality evolved largely concurrent with or after Malpighiaceae became restricted to Mexico (Figures 4, S5). We interpret these results to indicate that adaptations to precipitation seasonality largely arose *in situ* in Mexico as the abiotic conditions of SDTF were developing in this region (Figure S5). On average, the lag time for this *in situ* adaptation to precipitation seasonality was ~0.7 Myr. This pattern is supported by recent findings that identified a lag time in the adaptation of lineages to dry environments following their arrival into the Atacama Desert in South America (Guerrero et al., 2013).

Our results, however, provide a more nuanced perspective of *in situ* adaptation to dry forest environments and illuminate the timing of the origin and expansion of modern SDTF in Mexico. The first lineage to inhabit Mexico evolved dry forests adaptations ~26.0 Myr. For the remaining lineages, however, adaptation to precipitation seasonality did not arise until much later, around ~13.7 Myr on average. This suggests that although SDTF appears to have arisen in Mexico during the late Oligocene, it was likely geographically restricted at the time. It did not expand greatly until the mid-Miocene. This pattern is consistent with slow and steady north-south mountain building coinciding with the final uplifit of the Sierra Madre Occidental (34–15 Myr), followed by east-west orogeny of the Neovolcanic mountain chain (~23–2.5 Myr), which is hypothesized to have initiated the geological and abiotic conditions that gave rise to SDTF in Mexico (Moran-Zenteno, 1994; Becerra, 2005). Furthermore, this pattern more broadly coincides with global aridification that began during the Miocene, which marked the worldwide expansion of grasslands and succulent biomes (Cerling et al., 1993, 1997; Arakaki et al., 2011). Our results for Malpighiaceae further indicate that the expansion and widespread establishment of SDTF in the mid-Miocene marks a transition in the temporal pattern of adaptation to precipitation seasonality. While older lineages that become restricted to Mexico adapted to precipitation seasonality *in situ*, younger lineages that become restricted to Mexico (≤13.7 Myr) tended to be pre-adapted to precipitation seasonality (Figures 4, S5). These younger lineages appear to have arisen from ancestors that adapted to precipitation seasonality in Mexico and Central America, but subsequently became geographically restricted to Mexico. These patterns collectively illuminate the timing of the origin and expansion of modern SDTF in Mexico, and indicate that this biome more recently served as an important species pool for other SDTFs across the Neotropics.

It is important to keep in mind that the formation of the SDTF biome in Mexico occurred over millions of years, which appears to have afforded the earlier inhabitants of this region the time necessary to adapt to the novel abiotic conditions that define this biome today. If our hypothesis is correct, we should observe a pattern of more gradual adaptation to precipitation seasonality for dry periods that are consistent with intermediate historic levels (i.e., twice the level of current precipitation seasonality, 200 mm). When we perform this exercise, we observe a clear pattern of earlier adaption to these historic levels as expected under our hypothesis (Figures 4, S5). The extent to which this *in situ* adaptation occurred within the geographic footprint of the biome itself, vs. geographically adjacent regions in Mexico cannot be resolved with our data. However, we would expect *in situ* adaptation within the biome for many of these lineages. Further investigations using additional taxon sampling and far better geographic reconstructions of this biome throughout the Cenozoic would be required to determine this more confidently. Additionally, studies of recent range expansions and adaptions to novel environments could shed insight into this scenario. Nevertheless, the lag times we identify raise concerns about using modern biome categorizations as static characters for phylogenetic character state reconstruction (Davis et al., 2005; Crisp et al., 2009). In particular, our study indicates that ancestral reconstructions of biomes as static characters are problematic if the abiotic parameters that define these biomes today are much more recently evolved than the clades that inhabit them. Instead, our results suggest that inferring the evolution of key parameters that characterize these biomes (e.g., annual precipitation and precipitation seasonality) provide far more insight into the evolution of the lineages that inhabit the biome, as well as the biome itself.

## Conclusion

The assembly of a biome's flora depends on the complex interplay of dispersal, adaptation, and *in situ* diversification (Emerson and Gillespie, 2008). These processes in turn depend on both the geographic proximity and ecological similarity between the source pool and the biome in question (Emerson and Gillespie, 2008; Edwards and Donoghue, 2013). Our results additionally demonstrate that these processes are not static and change over evolutionary time. This has important implications for the composition of SDTF in Mexico to the formation of regional species pools and biomes more broadly. In the case of the formation of the Mexican Malpighiaceae flora, we found that changes in the geographic connectivity between South America and Mexico in the Miocene lead to dramatic changes in dispersal patterns from South America. Initially, lineages were limited to less frequent, longer-distance dispersal from South America. But increasingly the availability of Central America permitted more frequent, shorter-distance dispersal, facilitating vastly greater migration to Mexico starting in the mid-Miocene. The increased rate of dispersal, in turn, significantly influenced the composition of Mexican Malpighiaceae. While much of the extant species diversity in Mexico derives from *in situ* diversification, the overwhelming majority of phylogenetic diversity derives from multiple, independent introductions from South American ancestors spanning the phylogenetic breadth of the Malpighiaceae clade. Furthermore, climatic conditions that define the extant SDTF biome appear to have changed through time. While most lineages arrived in Mexico pre-adapted to one key factor of SDTF, total annual precipitation, these lineages subsequently adapted to another major axis, seasonal dry periods *in situ*. In this case, *in situ* adaption appears to have occurred gradually, over many millions of years, coincident with mountain building that established the geological conditions that maintain the biome today. Moreover, we demonstrate that once SDTF becomes widely established in Mexico, it increasingly becomes a source pool for other more recently developed SDTF, especially in Central America.

### Conflict of interest statement

The authors declare that the research was conducted in the absence of any commercial or financial relationships that could be construed as a potential conflict of interest.
