# Corrigendum to: The establishment of Central American migratory corridors and the biogeographic origins of seasonally dry tropical forests in Mexico

**DOI:** 10.3389/fgene.2015.00064

**Published:** 2015-02-24

**Authors:** Charles G. Willis, Brian F. Franzone, Zhenxiang Xi, Charles C. Davis

**Affiliations:** ^1^Harvard University Center for the EnvironmentCambridge, MA, USA; ^2^Department of Organismic and Evolutionary Biology, Harvard University HerbariaCambridge, MA, USA

**Keywords:** adaptive lag time, diversification, land bridge, long-distance dispersal, pre-adaptation, tropical biogeography, South America, species pool

In the original article, the title of the article in the Supplementary Material was wrong. The correct Supplementary Material appears below. This mistake does not change the scientific conclusions of the article in any way.

The author apologizes for this error.

## Conflict of interest statement

The authors declare that the research was conducted in the absence of any commercial or financial relationships that could be construed as a potential conflict of interest.

