# Supplementary material for: The establishment of Central American migratory corridors and the biogeographic origins of seasonally dry tropical forests in Mexico
Source: Front Genet. 2014 Dec 19;5:433. doi: 10.3389/fgene.2014.00433 (PMC4271706; doi:10.3389/fgene.2014.00433)
Supplement: Supplementary file 1 [file DataSheet1.DOC]

Supplementary Material:

**Community assembly meets biogeography: Malpighiaceae and the origin of seasonally dry tropical forest of Mexico**

Charles G. Willis,^1,2^ Brian Franzone, ^2^ Zhenxiang Xi, ^2^ and Charles C. Davis ^2^

^1^ Harvard University Center for the Environment, 24 Oxford Avenue, Cambridge, MA 02138, USA

^2^ Department of Organismic and Evolutionary Biology, Harvard University Herbaria, 22 Divinity Avenue, Cambridge, MA 02138, USA

**Table S1.** Newly sequenced taxa with voucher information and GenBank accession numbers. Herbarium acronyms follow Index herbariorum. Holmgren, P.K., Holmgren, N.H., and Barnett, L.C. (1990). *Part 1: The herbaria of the world*. New York, NY. New York Botanical Garden.

| Taxon | Voucher | Plastid | | | Nuclear |
| --- | --- | --- | --- | --- | --- |
|  |  | ***ndhF*** | ***matK*** | ***rbcL*** | ***PHYC*** |
| *Acridocarpus humbertii* Arènes | P. Phillipson 5269 (MICH) | KM197310 | KM197219 | KM197462 |  |
| *Adelphia macrophylla* (Rusby) W. R. Anderson | Timana & Jaramillo 2602 (MICH) | KM197311 | KM197220 |  |  |
| *Alicia macrodisca* (Triana & Planch.) W. R. Anderson | Nee 49368 (NY) | KM197312 |  | KM197463 |  |
| *Amorimia maritima* (Adr. Juss.) W. R. Anderson | Amorim et al. 2483 (MICH) | KM197313 | KM197221 |  |  |
| *Amorimia septentrionalis* W. R. Anderson | Pickel S. N. (US 1499453) | KM197314 | KM197222 |  |  |
| *Aspicarpa salicifolia* (Chodat) Nied. | Zardini and Guerrero 38669 (MICH) | KM197315 | KM197223 | KM197464 |  |
| *Aspicarpa schininii* W. R. Anderson | Arbo et al. 2651 (MICH) | KM197316 |  | KM197465 |  |
| *Aspidopterys elliptica* (Blume) Adr. Juss. | Ducke Res 4-557 (MICH) | KM197317 | KM197224 | KM197466 | KM197454 |
| *Banisteriopsis malifolia* (Nees & Mart.) B. Gates | Mori et al 21590 (MICH) | KM197318 | KM197225 | KM197467 | KM197455 |
| *Banisteriopsis anisandra* (Adr. Juss.) B. Gates | Kirkbride 4865 (MICH) | KM197319 | KM197226 | KM197468 |  |
| *Banisteriopsis basifixa* B. Gates | Anderson 13678 (MICH) | KM197320 | KM197227 |  |  |
| *Banisteriopsis campestris* (Adr. Juss.) Little | Irwin et al. 20354 (MICH) | KM197321 |  |  |  |
| *Banisteriopsis elegans* (Triana & Planch.) Sandwith | Albert de Escobar 3294 (MICH) | KM197322 | KM197228 | KM197469 |  |
| *Banisteriopsis goiana* B. Gates | Dias et al. 531 (MICH) | KM197323 | KM197229 |  |  |
| *Banisteriopsis megaphylla* (Adr. Juss.) B. Gates | Heringer et al. 6205 (MICH) | KM197324 | KM197230 |  |  |
| *Banisteriopsis padifolia* (Nied.) B. Gates | Matezki 320 (MICH) | KM197325 | KM197231 |  |  |
| *Banisteriopsis parviflora* (Adr. Juss.) B. Gates | Hatschbach 54117 (MICH) | KM197326 |  | KM197470 |  |
| *Banisteriopsis pulcherrima* (Sandwith) B. Gates | Maguire et al. 53669 (MICH) | KM197327 | KM197232 |  |  |
| *Banisteriopsis schizoptera* (Adr. Juss.) B. Gates | Anderson et al. 36421 (MICH) | KM197328 |  |  |  |
| *Banisteriopsis scutellata* (Griseb.) B. Gates | Kollmann 2511 (MICH) | KM197329 |  | KM197471 |  |
| *Banisteriopsis stellaris* (Griseb.) B. Gates | Santos et al. 673 (MICH) | KM197330 | KM197233 | KM197472 |  |
| *Banisteriopsis vernoniifolia* (Adr. Juss.) B. Gates | Cavalcanti et al. 1288 (MICH) | KM197331 | KM197234 |  |  |
| *Bronwenia acapulcensis* (Rose) W. R. Anderson & C. Davis | Burnham 977 (MICH) | KM197332 | KM197235 | KM197473 |  |
| *Bronwenia megaptera* (B. Gates) W. R. Anderson & C. Davis | Noblick and Lemos 2754 (MICH) | KM197333 | KM197236 |  |  |
| *Bronwenia wurdackii* (B. Gates) W. R. Anderson & C. Davis | Baker 6145 (MICH) | KM197334 | KM197237 | KM197474 |  |
| *Bunchosia armeniaca* (Cav.) DC. | Nee 41995 (MICH) |  | KM197238 |  |  |
| *Bunchosia biocellata* Schltdl. | Acosta & Dorantes 217 (MICH) | KM197335 |  |  |  |
| *Bunchosia caroli* W. R. Anderson | Torres 5426 (MICH) | KM197336 | KM197239 |  |  |
| *Bunchosia cestrifolia* Cuatrec. | Flora Falcon 589 (MICH) | KM197337 | KM197240 |  |  |
| *Bunchosia cestrifolia* Cuatrec. | Van der Werff 3567 (MICH) |  | KM197241 |  |  |
| *Bunchosia cruciana* | Ventura 11680 (MICH) | KM197338 | KM197242 |  |  |
| *Bunchosia hookeriana* Adr. Juss. | Clark 2955 (MICH) | KM197339 |  |  |  |
| *Bunchosia lindeniana* Adr. Juss. | Nee 23790 (MICH) | KM197340 | KM197243 |  |  |
| *Bunchosia luzmariae* W. R. Anderson | Tellez 8289 (MICH) | KM197341 | KM197244 |  |  |
| *Bunchosia maritima* (Vell.) J. F. Macbr. | Gentry & Zardini 49722 (MICH) | KM197342 |  |  |  |
| *Bunchosia matudae* Lundell | McVaugh 22351 (MICH) | KM197343 | KM197245 |  |  |
| *Bunchosia montana* Adr. Juss. | Torres 6970 (MICH) |  | KM197246 |  |  |
| *Bunchosia nitida* (Jacq.) DC. | Murray & Johnson 1445 (MICH) | KM197344 | KM197247 |  |  |
| *Bunchosia pallescans* | Amorim 3281 (MICH) | KM197345 | KM197248 |  |  |
| *Bunchosia palmeri* S. Watson | Tellez 12664 (MICH) | KM197346 | KM197249 |  |  |
| *Bunchosia paraguariensis* Nied. | Nee 38583 (MICH) | KM197347 | KM197250 |  |  |
| *Bunchosia pernambucana* W. R. Anderson | Tscha et al. 15 (MICH) | KM197348 | KM197251 |  |  |
| *Bunchosia plowmanii* W. R. Anderson | Neill & Nunez 10496 (MICH) | KM197349 |  |  |  |
| *Bunchosia pringlei* S. Watson | Ventura 3659 (MICH) | KM197350 | KM197252 |  |  |
| *Bunchosia pseudonitida* Cuatrec. | Silverstone-Sopkin 3433 (MICH) | KM197351 | KM197253 |  |  |
| *Bunchosia strigosa* Schltdl. | Reyes 1053 (MICH) | KM197352 | KM197254 |  |  |
| *Bunchosia ststyca* | DuivenVoorden et al. 138 (MICH) | KM197353 | KM197255 |  |  |
| *Bunchosia velutina* | Laughlin 879 (MICH) | KM197354 |  |  |  |
| *Calcicola sericea* (Nied.) W. R. Anderson & C. Davis | Tenorio 6356 (MICH) | KM197355 | KM197256 | KM197475 |  |
| *Callaeum coactum* D. M. Johnson | Anderson and Laskowski 4299 (MICH) | KM197356 | KM197257 | KM197476 |  |
| *Callaeum macropterum* (DC.) D. M. Johnson | Anderson and Laskowski 4556 (MICH) | KM197357 | KM197258 | KM197477 |  |
| *Camarea affinis* A. St.-Hil. | Pirani 1557 (MICH) | KM197358 |  |  |  |
| *Caucanthus albidus* Nied. | R. G. Wieland 1069 (MO) | KM197359 | KM197259 |  |  |
| *Caucanthus edulis* Forssk. | DeWilde 5920 (M) | KM197360 | KM197260 |  |  |
| *Christianella mesoamericana* (W. R. Anderson) W. R. Anderson | Standley 87257 (F) | KM197361 |  | KM197478 |  |
| *Cottsia linearis* (Wiggins) W. R. Anderson & C. Davis | WRA 12540 | KM197395 | KM197279 | KM197486 | KM197457 |
| *Dicella conwayi* Rusby | Krukoff 10298 (MICH) | KM197362 | KM197261 | KM197479 |  |
| *Diplopterys amplectens* (B. Gates) W. R. Anderson & C. Davis | Carvalho et al. 4032 (MICH) | KM197363 |  |  |  |
| *Diplopterys leiocarpa* (Adr. Juss.) W. R. Anderson & C. Davis | Sanchez Vega 3710 (MICH) | KM197364 | KM197262 |  |  |
| *Diplopterys longialata* (Nied.) W. R. Anderson & C. Davis | Krukoff 10022 (MICH) | KM197365 | KM197263 |  |  |
| *Diplopterys rondoniensis* (B. Gates) W. R. Anderson & C. Davis | Nee 34750 (MICH) | KM197366 |  |  |  |
| *Echinopterys setosa* Brandegee | Muller 3281 (MICH) | KM197367 | KM197264 |  |  |
| *Excentradenia propinqua* (W. R. Anderson) W. R. Anderson | Linderman et al. 51 (MICH) | KM197368 |  |  |  |
| *Galphimia angustifolia* Benth. | Daniel 2393 (MICH) | KM197369 |  |  |  |
| *Galphimia australis* Chodat | Anderson 12362 (MICH) | KM197370 | KM197265 | KM197480 |  |
| *Galphimia elegans* Baill. | Conzatti 4854 (MICH) | KM197371 |  |  |  |
| *Galphimia glauca* Cav. | Servin 268 (MICH) | KM197372 |  |  |  |
| *Galphimia langlassei* (S. F. Blake) C. E. Anderson | McVaugh 10324 (MICH) | KM197373 |  |  |  |
| *Galphimia oaxacana* C. E. Anderson | Torres 5880 (MICH) | KM197374 |  |  |  |
| *Galphimia* sp*.* | Garcia 2444 (MICH) | KM197375 |  |  |  |
| *Galphimia vestita* S. Watson | White 3574 (MICH) | KM197376 |  | KM197481 |  |
| *Gaudichaudia cycloptera* (DC.) W. R. Anderson | Anderson & Laskowski 4545 (MICH) | KM197377 | KM197266 |  |  |
| *Gaudichaudia diandra* (Nied.) Chodat | Jessup 4034 (MICH) | KM197378 | KM197267 |  |  |
| *Gaudichaudia galeottiana* (Nied.) Chodat | Anderson & Laskowski 4276 (MICH) | KM197379 | KM197268 |  |  |
| *Gaudichaudia subverticillata* Rose | Anderson & Anderson 5104 (MICH) | KM197380 | KM197269 |  |  |
| *Heladena bunchosioides* (Adr. Juss.) Adr. Juss. | Folli 4653 (MICH) | KM197381 | KM197270 | KM197482 |  |
| *Heteropterys cordifolia* Adr. Juss. | A. Amorim 3095 (MICH) | KM197382 |  | KM197483 |  |
| *Heteropterys glazioviana* Nied. | A. Amorim 3318 (MICH) | KM197383 |  |  |  |
| *Heteropterys laurifolia* (L.) Adr. Juss. | McVaugh 22431 (MICH) | KM197384 | KM197271 |  |  |
| *Heteropterys lindeniana* Adr. Juss. | Whitefoord 8357 (MICH) | KM197385 |  |  |  |
| *Heteropterys palmeri* Rose | Daniel 3284 (MICH) | KM197386 | KM197272 |  |  |
| *Hiptage stellulifera* Arènes | A. F. G. Kerr 17280 (P) | KM197387 | KM197273 |  |  |
| *Hiptage calcicola* Sirirugsa | D. J. Middleton et al. 1155 | KM197388 |  |  |  |
| *Hiptage poilanei* Arènes | L. Averyanou et al VH1401 (P) | KM197389 | KM197274 | KM197484 |  |
| *Hiptage* sp. | K. Larsen, S.S. Larsen 33641 (P) | KM197390 |  |  |  |
| *Hiptage* sp. | C. F. Van Beusekom and T. Santisuk 2705 (P) | KM197391 | KM197275 |  |  |
| *Hiraea barclayana* Benth. | Flores 1859 (MICH) | KM197392 | KM197276 |  |  |
| *Hiraea reclinata* Blanco | Balick 1925 (MICH) | KM197393 | KM197277 |  |  |
| *Janusia guaranitica* (A. St.-Hil.) Adr. Juss. | Schinini 13291B | KM197394 | KM197278 | KM197485 | KM197456 |
| *Janusia prancei* W. R. Anderson | Janssen and Gemtchujnicov 356 (MICH) | KM197396 | KM197280 | KM197487 |  |
| *Janusia schwannioides* W. R. Anderson | Mori et al. 9517 (MICH) | KM197397 |  | KM197488 |  |
| *Malpighia galeottiana* Adr. Juss. | Pringle 7472 (MICH) | KM197398 | KM197281 |  |  |
| *Malpighia lundellii* C. V. Morton | Gentle 885 (MICH) | KM197399 |  |  |  |
| *Malpighia* sp. | AP2 (K) | KM197400 | KM197282 | KM197489 | KM197458 |
| *Malpighia wendtii* W. R. Anderson | Contreras 8841 (MICH) | KM197401 | KM197283 |  |  |
| *Malpighiodes guianensis* (W. R. Anderson) W. R. Anderson | Pipoly 7599 (MICH) | KM197402 | KM197284 | KM197490 |  |
| *Mascagnia almedae* W. R. Anderson | Breedlove 56949 (MICH) | KM197403 | KM197285 |  |  |
| *Mascagnia dissimilis* C. V. Morton & Moldenke | Anderson 1225 (MICH) | KM197405 | KM197287 |  |  |
| *Mascagnia eggersiana* (Nied.) W. R. Anderson | Schunke V. 3822 (MICH) | KM197406 | KM197288 | KM197492 |  |
| *Mascagnia ovatifolia* (H. B. K.) Griseb. | Martinez 43 (MICH) | KM197407 |  |  |  |
| *Mascagnia schnunkei* | Schunke 12533 (MICH) | KM197408 |  |  |  |
| *Mascagnia strigulosa* (Rusby) Nied. | Gentry 13318 (MICH) | KM197409 |  |  |  |
| *Mezia angelica* W. R. Anderson | Mori et al. 20945 (MICH) | KM197410 | KM197289 | KM197493 |  |
| *Mezia beckii* W. R. Anderson | Jardim 2397 (MICH) | KM197411 | KM197290 |  |  |
| *Mezia mariposa* W. R. Anderson | Krukoff 5452 (MICH) | KM197412 | KM197291 |  |  |
| *Mezia tomentosa* W. R. Anderson | Gudino 396 (MICH) | KM197413 | KM197292 | KM197494 |  |
| *Microsteira amphiamensis* | Rabesandratana 4195 (P) | KM197414 | KM197293 |  |  |
| *Microsteira argyrophylla* (Adr. Juss.) Dubard & Dop | Lorence 1905 (P) | KM197415 |  | KM197495 |  |
| *Microsteira curtisii* Baker | G. Cremers 2827 (P) | KM197416 |  |  |  |
| *Microsteira paniculata* Arènes | Ramarokoto 5043 RN | KM197417 |  |  |  |
| *Microsteira* sp. | M. Luckow 4216 (MO 5794317) | KM197418 | KM197294 |  |  |
| *Microsteira* sp. | N. Dumetz et al. 749 (P) | KM197419 | KM197295 |  |  |
| *Peixotoa bahiana* C. E. Anderson | Irwin et al. 14889 (MICH) | KM197420 |  |  |  |
| *Peixotoa hispidula* Adr. Juss. | Jardim 593 (MICH) | KM197421 | KM197296 |  |  |
| *Peixotoa tomentosa* Adr. Juss. | Harley et al. CFCR 5897 (MICH) | KM197422 |  |  |  |
| *Psychopterys dipholiphylla* (Small) W. R. Anderson & S. Corso | WRA 13816 | KM197404 | KM197286 | KM197491 | KM197459 |
| *Psychopterys mcvaughii* W. R. Anderson & S. Corso | McVaugh 23243 (MICH) | KM197423 |  |  |  |
| *Psychopterys rivularis* (C. V. Morton & Standl.) W. R. Anderson & S. Corso | Ibarra 603 (MICH) | KM197424 |  |  |  |
| *Ryssopterys* sp. | M. Mackee 14932 (P) | KM197427 |  |  |  |
| *Ryssopterys* sp. | Motley & Cameron 2215 (NY) | KM197428 | KM197297 | KM197498 | KM197460 |
| *Spachea membranacea* Cuatrec. | Foster 873 (MICH) | KM197429 |  | KM197499 |  |
| *Spachea tricarpa* Adr. Juss. | Rimachi 3201 (NY) | KM197430 | KM197298 | KM197500 |  |
| *Sphedamnocarpus galphimiifolius* (Adr. Juss.) Szyszyl. | Jo'burg BG 1/88 (K) | KM197431 | KM197299 | KM197501 | KM197461 |
| *Sphedamnocarpus isaloensis* | P.B. Phillipson et al. (MICH) | KM197432 |  |  |  |
| *Stigmaphyllon angustifolium* (Nied.) C. E. Anderson | G. McPherson 5316 (P) | KM197425 |  | KM197496 |  |
| *Stigmaphyllon cuzcanum* C. E. Anderson | Galiano and Suclli 5689 (MICH) | KM197433 | KM197300 | KM197502 |  |
| *Stigmaphyllon discolor* (Gand.) C. E. Anderson | G. McPherson 2266 (P) | KM197426 |  | KM197497 |  |
| *Stigmaphyllon ellipticum* (H. B. K.) Adr. Juss. | Ventura 2624 (MICH) | KM197434 | KM197301 |  |  |
| *Stigmaphyllon pseudopuberum* Nied. | Breedlove 44392 (MICH) | KM197435 |  |  |  |
| *Stigmaphyllon retusum* Griseb. | Gentry 12285 (MICH) | KM197436 |  |  |  |
| *Stigmaphyllon selerianum* Nied. | Salinas T. 6680 (MICH) | KM197437 | KM197302 |  |  |
| *Tetrapterys ambigua* (Adr. Juss.) Nied. | Krapovickas et al. 46063 (MICH) | KM197438 |  | KM197503 |  |
| *Tetrapterys argentea* Bertol. | Matuda 5085 (MICH) | KM197439 |  |  |  |
| *Tetrapterys cardiophylla* Nied. | Ganev 504 (MICH) |  | KM197303 |  |  |
| *Tetrapterys fimbripetala* Adr. Juss. | Huber and Gorzula 11154 (MICH) | KM197440 |  |  |  |
| *Tetrapterys heterophylla* (Griseb.) W. R. Anderson | McDougall 1970 (MICH) | KM197441 |  |  |  |
| *Tetrapterys maranhamensis* Adr. Juss. | Pires and Silva 1759 (MICH) | KM197442 |  |  |  |
| *Tetrapterys mexicana* Hook. & Arn. | Anderson 5997 (MICH) | KM197443 |  |  |  |
| *Tetrapterys mucronata* Cav. | Dubs 1684 (MICH) | KM197444 | KM197304 | KM197504 |  |
| *Tetrapterys paludosa* Adr. Juss. | Arbo et al. 5465 (MICH) |  | KM197305 |  |  |
| *Tetrapterys pusilla* Steyerm. | Maguire 32201 (MICH) | KM197445 |  |  |  |
| *Tetrapterys seleriana* Nied. | Cabera 4891 (MICH) | KM197446 | KM197306 |  |  |
| *Tetrapterys styloptera* Adr. Juss. | Beck 21628 (MICH) | KM197447 |  | KM197505 |  |
| *Tetrapterys vacciniifolia* Adr. Juss. | Wingfield 13017 (MICH) | KM197448 | KM197307 |  |  |
| *Thryallis laburnum* S. Moore | Gentry et al. 73895 (MICH) | KM197449 | KM197308 | KM197506 |  |
| *Thryallis parviflora* C. E. Anderson | Heringer 14262 (MICH) | KM197450 |  |  |  |
| *Triaspis odorata* (Willd.) Adr. Juss. | C.C. Davis 99-20 | KM197451 | KM197309 | KM197507 |  |
| *Tristellateia australasiae* A. Rich. | Kit Yock Chan 1358 (A) | KM197452 |  |  |  |
| *Tristellateia* sp. | Rabevohitra et al. 4355 (P) | KM197453 |  |  |  |

**Table S2.** Biogeographic range data from Anderson (personal communication; 2006 onwards) and precipitation data from the World Bioclim dataset at 30” resolution (www.worldclim.org). Region codes are: SA – South America; Ca – Caribbean; CA – Central America; Me – Mexico; Af – Africa; Ma – Madagascar; As – Asia. Bracketed regions indicate taxa that inhabit multiple regions.

| **Taxa** | **Region** | **Total Annual Precipitation (mm)** | **Precipitation Seasonality (mm)** |
| --- | --- | --- | --- |
| *Acmanthera latifolia* | SA | 2301.5 | 266 |
| *Acridocarpus adenophorus* | Ma | 2189.1 | 260.7 |
| *Acridocarpus alternifolius* | Af | 1579.3 | 140.1 |
| *Acridocarpus chevalieri* | Af | 1227 | 59.4 |
| *Acridocarpus excelsus* | Ma | 1271.2 | 35.6 |
| *Acridocarpus humbertii* | Ma | 540 | 13.9 |
| *Acridocarpus macrocalyx* | Af | 2018 | 65 |
| *Acridocarpus natalitius* | Af | 881.4 | 73.3 |
| *Acridocarpus orientalis* | Af | NA | NA |
| *Acridocarpus scheffleri* | Af | NA | NA |
| *Acridocarpus smeathmannii* | Af | 1706.8 | 93.6 |
| *Acridocarpus spectabilis* | Af | 1172.8 | 8.9 |
| *Acridocarpus staudtii* | Af | 2402.2 | 109.5 |
| *Acridocarpus zanzibaricus* | Af | 1024.2 | 74.7 |
| *Adelphia hiraea* | SA/Ca/CA/Me | 2694.9 | 204.7 |
| *Adelphia macrophylla* | SA | 1938.3 | 213.4 |
| *Aenigmatanthera doniana* | SA | NA | NA |
| *Aenigmatanthera lasiandra* | SA | 1484.4 | 64.7 |
| *Alicia anisopetala* | SA | 1618.6 | 224.1 |
| *Alicia macrodisca* | SA | 1942.6 | 189.7 |
| *Amorimia amazonica* | SA | 1557.8 | 182.9 |
| *Amorimia exotropica* | SA | NA | NA |
| *Amorimia kariniana* | SA | 978 | 3 |
| *Amorimia maritima* | SA | NA | NA |
| *Amorimia rigida* | SA | 1117.5 | 158 |
| *Amorimia septentrionalis* | SA | NA | NA |
| *Amorimia velutina* | SA | NA | NA |
| *Aspicarpa brevipes* | Me | 1006 | 19.6 |
| *Aspicarpa harleyi* | SA | 786 | 43.1 |
| *Aspicarpa hirtella* | Me | 614.2 | 28.4 |
| *Aspicarpa hyssopifolia* | Me | 503.3 | 47 |
| *Aspicarpa pulchella* | SA | 1541.1 | 284 |
| *Aspicarpa salicifolia* | SA | 1480.8 | 277.8 |
| *Aspicarpa schininii* | SA | 1111.5 | 119.5 |
| *Aspicarpa sericea* | SA | 853.1 | 74 |
| *Aspidopterys tomentosa* | As | 1846 | 334 |
| *Banisteriopsis acerosa* | SA | 1421 | 156.5 |
| *Banisteriopsis adenopoda* | SA | 1485.5 | 111 |
| *Banisteriopsis angustifolia* | SA | 1191.9 | 38.2 |
| *Banisteriopsis anisandra* | SA | 1449.6 | 36.5 |
| *Banisteriopsis argyrophylla* | SA | 1480.2 | 76.6 |
| *Banisteriopsis basifixa* | SA | NA | NA |
| *Banisteriopsis caapi* | SA | 2870.4 | 441.2 |
| *Banisteriopsis calcicola* | SA | 874 | 3.5 |
| *Banisteriopsis campestris* | SA | 1409.7 | 53.3 |
| *Banisteriopsis confusa* | SA | 1581.6 | 92.8 |
| *Banisteriopsis elegans* | SA/CA/Me | 3884.1 | 469 |
| *Banisteriopsis gardneriana* | SA | 1603.5 | 57.5 |
| *Banisteriopsis goiana* | SA | NA | NA |
| *Banisteriopsis harleyi* | SA | 926.8 | 82.7 |
| *Banisteriopsis laevifolia* | SA | 1408.8 | 47.6 |
| *Banisteriopsis latifolia* | SA | 1619.4 | 25.6 |
| *Banisteriopsis martiniana* | SA | 2639.8 | 434.9 |
| *Banisteriopsis megaphylla* | SA | 1538.7 | 30 |
| *Banisteriopsis muricata* | SA/CA/Me | 1859 | 127.7 |
| *Banisteriopsis nummifera* | SA | 1537.1 | 140.6 |
| *Banisteriopsis padifolia* | SA | 1961 | 275 |
| *Banisteriopsis paraguariensis* | SA | 1631 | 268 |
| *Banisteriopsis parviflora* | SA | 958.8 | 99.8 |
| *Banisteriopsis prancei* | SA | 1886.1 | 95.9 |
| *Banisteriopsis pulcherrima* | SA | 2088.1 | 222.2 |
| *Banisteriopsis pulchra* | SA | 1483 | 142.4 |
| *Banisteriopsis schizoptera* | SA | 1309.7 | 29.1 |
| *Banisteriopsis schwannioides* | SA | 2423 | 260 |
| *Banisteriopsis scutellata* | SA | NA | NA |
| *Banisteriopsis sellowiana* | SA | 1249 | 165 |
| *Banisteriopsis stellaris* | SA | 1353.2 | 48 |
| *Banisteriopsis vernoniifolia* | SA | 1709.3 | 22.3 |
| *Barnebya dispar* | SA | NA | NA |
| *Blepharandra fimbriata* | SA | 2886.3 | 228.3 |
| *Blepharandra heteropetala* | SA | 3112.1 | 378.1 |
| *Blepharandra hypoleuca* | SA | 2135.3 | 220.7 |
| *Brachylophon curtisii* | As | NA | NA |
| *Bronwenia acapulcensis* | SA/CA/Me | NA | NA |
| *Bronwenia cinerascens* | SA | 1131.9 | 97.9 |
| *Bronwenia cornifolia* | SA/CA/Me | 2026 | 139.2 |
| *Bronwenia ferruginea* | SA | NA | NA |
| *Bronwenia mathiasiae* | SA | 2192.3 | 203 |
| *Bronwenia megaptera* | SA | 869 | 46 |
| *Bronwenia wurdackii* | SA/CA | 2275.7 | 228.4 |
| *Bunchosia angustifolia* | SA | 1586.2 | 159.8 |
| *Bunchosia apiculata* | SA | 2319.7 | 181.3 |
| *Bunchosia armeniaca* | SA | 2300.5 | 316.1 |
| *Bunchosia biocellata* | CA/Me | 1341.3 | 82 |
| *Bunchosia caroli* | Me | 1198 | 24 |
| *Bunchosia cestrifolia* | SA | 1141 | 73 |
| *Bunchosia decussiflora* | SA | 2378.2 | 261.9 |
| *Bunchosia deflexa* | SA | NA | NA |
| *Bunchosia glandulifera* | SA | 2218.6 | 254.8 |
| *Bunchosia glandulosa* | Ca | 1164.2 | 96.9 |
| *Bunchosia hookeriana* | SA | 2218.6 | 320.6 |
| *Bunchosia lindeniana* | CA/Me | 2108.1 | 151 |
| *Bunchosia luzmariae* | Me | NA | NA |
| *Bunchosia macrophylla* | SA/CA/Me | 3256.7 | 284.1 |
| *Bunchosia maritima* | SA | 1653.7 | 180.3 |
| *Bunchosia matudae* | CA/Me | 1605.5 | 27 |
| *Bunchosia mcvaughii* | Me | 785.5 | 11.6 |
| *Bunchosia mollis* | SA | 1553.1 | 96.4 |
| *Bunchosia montana* | CA/Me | 906 | 25.6 |
| *Bunchosia nitida* | SA/CA/Me | 2466.2 | 122.4 |
| *Bunchosia odorata* | SA/CA | 2260.3 | 92.9 |
| *Bunchosia pallescens* | SA | 1666.6 | 295.4 |
| *Bunchosia palmeri* | Me | 1032.4 | 22.8 |
| *Bunchosia paraguariensis* | SA | 1514.2 | 117.9 |
| *Bunchosia pernambucana* | SA | NA | NA |
| *Bunchosia pilocarpa* | SA | 1178.2 | 113.2 |
| *Bunchosia plowmannii* | SA | 917.5 | 7 |
| *Bunchosia polystachia* | Ca/CA | 3065.9 | 247.8 |
| *Bunchosia pringlei* | Me | 888 | 52 |
| *Bunchosia pseudonitida* | SA | 1661.7 | 208.5 |
| *Bunchosia strigosa* | Me | 899.3 | 24.7 |
| *Bunchosia swartziana* | Ca/CA/Me | 1210.5 | 101.4 |
| *Bunchosia systyla* | SA | NA | NA |
| *Bunchosia ternata* | CA | 3266.1 | 293.9 |
| *Bunchosia veluticarpa* | CA | 2966.6 | 199.1 |
| *Burdachia sphaerocarpa* | SA | 2477.9 | 311.8 |
| *Byrsonima basiloba* | SA | 1514.3 | 23.6 |
| *Byrsonima coccolobifolia* | SA | 1444.1 | 85.6 |
| *Byrsonima crassifolia* | SA/Ca/CA/Me | 1908.3 | 115.1 |
| *Byrsonima crispa* | SA | 2660.3 | 222.3 |
| *Byrsonima duckeana* | SA | 2129.8 | 241.6 |
| *Byrsonima lucida* | Ca | 1620.6 | 174.1 |
| *Byrsonima macrophylla* | SA | 1486.6 | 33.9 |
| *Byrsonima morii* | SA | 890.3 | 75.7 |
| *Byrsonima triopterifolia* | SA | 935.4 | 82.4 |
| *Calcicola parvifolia* | Me | 681.4 | 28.2 |
| *Calcicola sericea* | Me | 309.3 | 21.3 |
| *Callaeum antifebrile* | SA | 2446.9 | 317.6 |
| *Callaeum clavipetalum* | Me | NA | NA |
| *Callaeum coactum* | Me | 904.1 | 13 |
| *Callaeum macropterum* | Me | 603.1 | 26.1 |
| *Callaeum malpighioides* | CA/Me | 1653.5 | 116.8 |
| *Callaeum nicaraguense* | CA/Me | 1319.5 | 25.4 |
| *Callaeum psilophyllum* | SA | 1395.3 | 183.1 |
| *Callaeum septentrionale* | Me | 857.2 | 50.7 |
| *Camarea affinis* | SA | 1462.7 | 64.4 |
| *Camarea axillaris* | SA | 965.1 | 57.6 |
| *Carolus anderssonii* | SA | 1020.8 | 57.5 |
| *Carolus chasei* | SA | 729.5 | 80 |
| *Carolus chlorocarpus* | SA | 1227.2 | 130.5 |
| *Carolus sinemariensis* | SA/CA/Me | 2273.1 | 149.3 |
| *Caucanthus auriculatus* | Af | 768.6 | 32.3 |
| *Christianella mesoamericana* | CA/Me | 2874 | 102.5 |
| *Christianella multiglandulosa* | SA | 1518 | 127 |
| *Christianella surinamensis* | SA | 1803.1 | 138.9 |
| *Coleostachys genipifolia* | SA | 2391 | 238 |
| *Cordobia argentea* | SA | 571.6 | 26.8 |
| *Diacidia ferruginea* | SA | 3129 | 237.5 |
| *Diacidia galphimioides* | SA | 2948.2 | 356.6 |
| *Dicella bracteosa* | SA | 1047.3 | 147.7 |
| *Dicella conwayi* | SA | 1801.2 | 160.7 |
| *Dicella julianii* | SA | 2989.6 | 488.4 |
| *Dicella macroptera* | SA | 1385.7 | 89.4 |
| *Dicella nucifera* | SA | 1590.4 | 287.6 |
| *Digoniopterys microphylla* | Ma | 391 | 18.3 |
| *Dinemagonum gayanum* | SA | 54 | 0.5 |
| *Dinemandra ericoides* | SA | 73.1 | 2.8 |
| *Diplopterys amplectens* | SA | 1054 | 3 |
| *Diplopterys cabrerana* | SA | 2817.3 | 476.2 |
| *Diplopterys hypericifolia* | SA | 1398.8 | 27.4 |
| *Diplopterys leiocarpa* | SA | 791 | 80.5 |
| *Diplopterys longialata* | SA | 2060.7 | 245.3 |
| *Diplopterys lutea* | SA | 1203.3 | 95.8 |
| *Diplopterys nutans* | SA/CA | 2360.9 | 206.2 |
| *Diplopterys pubipetala* | SA | 1552.8 | 90.4 |
| *Diplopterys rondoniensis* | SA | 1572 | 51 |
| *Diplopterys valvata* | SA | 954 | 6.5 |
| *Diplopterys virgultosa* | SA | 1054 | 3 |
| *Echinopterys eglandulosa* | Me | 651.6 | 17 |
| *Echinopterys setosa* | Me | 325.8 | 26.8 |
| *Ectopopterys soejartoi* | SA | 3236.9 | 617.6 |
| *Excentradenia propinqua* | SA | 1961 | 166.5 |
| *Flabellaria paniculata* | Af | 1634.8 | 84.8 |
| *Flabellariopsis acuminata* | Af | 1688.5 | 73.5 |
| *Gallardoa fischeri* | SA | 674 | 54 |
| *Galphimia angustifolia* | Me | 478.3 | 37.4 |
| *Galphimia australis* | SA | 1098.8 | 139.7 |
| *Galphimia brasiliensis* | SA | 1344.3 | 232.7 |
| *Galphimia elegans* | Me | 704.8 | 14.3 |
| *Galphimia glandulosa* | Me | 1095.1 | 21.3 |
| *Galphimia glauca* | Me | 880.8 | 37.2 |
| *Galphimia gracilis* | CA/Me | 1411.9 | 87.4 |
| *Galphimia langlassei* | Me | 1289 | 20 |
| *Galphimia mexiae* | Me | NA | NA |
| *Galphimia mirandae* | Me | NA | NA |
| *Galphimia multicaulis* | Me | 1000.2 | 30.8 |
| *Galphimia oaxacana* | Me | 722 | 7.3 |
| *Galphimia platyphylla* | SA | 1624.3 | 277.3 |
| *Galphimia vestita* | Me | 705.3 | 28.5 |
| *Gaudichaudia albida* | SA/CA/Me | 1296.6 | 57.5 |
| *Gaudichaudia cycloptera* | Me | 1406.3 | 37 |
| *Gaudichaudia cynanchoides* | Me | 795.8 | 28.5 |
| *Gaudichaudia diandra* | Me | NA | NA |
| *Gaudichaudia galeottiana* | Me | 679.3 | 16.3 |
| *Gaudichaudia hexandra* | CA | 2060.5 | 57.8 |
| *Gaudichaudia krusei* | Me | 1333 | 23.6 |
| *Gaudichaudia mcvaughii* | Me | 922.6 | 15.8 |
| *Gaudichaudia subverticillata* | Me | 869 | 27 |
| *Glandonia macrocarpa* | SA | 2560.5 | 411.5 |
| *Heladena bunchosioides* | SA | NA | NA |
| *Heladena multiflora* | SA | 1634.9 | 294.6 |
| *Henleophytum echinatum* | Ca | NA | NA |
| *Heteropterys aureosericea* | SA | 2921.9 | 537.6 |
| *Heteropterys bahiensis* | SA | NA | NA |
| *Heteropterys bicolor* | SA | 1302.7 | 240.5 |
| *Heteropterys brachiata* | SA/CA/Me | 1482 | 87.7 |
| *Heteropterys byrsonimifolia* | SA | 1330.2 | 35.7 |
| *Heteropterys capixaba* | SA | NA | NA |
| *Heteropterys catingarum* | SA | 1353.5 | 85.5 |
| *Heteropterys chrysophylla* | SA | 1020.3 | 105.3 |
| *Heteropterys ciliata* | SA | NA | NA |
| *Heteropterys conformis* | SA | 1035 | 160 |
| *Heteropterys cordifolia* | SA | 1596.3 | 333 |
| *Heteropterys dumetorum* | SA | 918.8 | 55 |
| *Heteropterys glazioviana* | SA | NA | NA |
| *Heteropterys imperata* | SA | 1631.1 | 284.8 |
| *Heteropterys laurifolia* | SA/Ca/CA/Me | 1888.1 | 108 |
| *Heteropterys leona* | SA/CA/Af | 3569.8 | 426.1 |
| *Heteropterys leschenaultiana* | SA | 1305.8 | 236 |
| *Heteropterys lindeniana* | CA/Me | 1633.7 | 125 |
| *Heteropterys lindleyana* | SA | 1597.5 | 142 |
| *Heteropterys macrostachya* | SA/CA/Me | 2523.4 | 221.2 |
| *Heteropterys megaptera* | SA | 1764 | 251.7 |
| *Heteropterys nitida* | SA | 1312.4 | 109.6 |
| *Heteropterys nordestina* | SA | 1449.1 | 223.9 |
| *Heteropterys palmeri* | CA/Me | 847 | 15.8 |
| *Heteropterys pauciflora* | SA | 1719 | 369 |
| *Heteropterys pteropetala* | SA | 1460.1 | 50.4 |
| *Heteropterys rhopalifolia* | SA | 1037.3 | 46.3 |
| *Heteropterys rufula* | SA | NA | NA |
| *Heteropterys sanctorum* | SA | 1284 | 253 |
| *Heteropterys sericea* | SA | 1040.6 | 89.2 |
| *Heteropterys sincorensis* | SA | 884 | 59 |
| *Heteropterys steyermarkii* | SA | 2943.7 | 202.3 |
| *Heteropterys ternstroemiifolia* | SA | 1474 | 101 |
| *Heteropterys trichanthera* | SA | 746.1 | 47.5 |
| *Hiptage benghalensis* | As | 2539.4 | 187.6 |
| *Hiptage calcicola* | As | NA | NA |
| *Hiptage candicans* | As | NA | NA |
| *Hiptage detergens* | As | NA | NA |
| *Hiptage poilanei* | As | NA | NA |
| *Hiptage stellulifera* | As | NA | NA |
| *Hiraea barclayana* | CA/Me | 1287.2 | 43.8 |
| *Hiraea fagifolia* | SA/CA/Me | 2527.1 | 299.8 |
| *Hiraea reclinata* | SA/CA/Me | 1883 | 106.2 |
| *Hiraea smilacina* | SA/CA/Me | 3352.7 | 257.8 |
| *Hiraea wiedeana* | SA | NA | NA |
| *Janusia anisandra* | SA | 797.7 | 26.8 |
| *Janusia christianeae* | SA | NA | NA |
| *Janusia guaranitica* | SA | 1200.8 | 162.7 |
| *Janusia hexandra* | SA | 1118 | 199 |
| *Janusia janusioides* | SA | 2404 | 94 |
| *Janusia linearifolia* | SA | NA | NA |
| *Janusia mediterranea* | SA | 1617 | 271 |
| *Janusia prancei* | SA | 2192 | 131 |
| *Janusia schwannioides* | SA | 730.3 | 14.7 |
| *Jubelina riparia* | SA | 3184 | 184 |
| *Jubelina rosea* | SA | 2651.3 | 257.8 |
| *Jubelina uleana* | SA | 3206.4 | 612.2 |
| *Jubelina wilburii* | SA/CA | 3291.9 | 346.7 |
| *Lophanthera hammelii* | CA | 3843.7 | 330.5 |
| *Lophanthera lactescens* | SA | 2199 | 223 |
| *Lophanthera longifolia* | SA | 3034.3 | 462.2 |
| *Lophanthera pendula* | SA | 3072 | 589 |
| *Lophopterys floribunda* | SA | 2576 | 133.7 |
| *Lophopterys inpana* | SA | 1931.4 | 125.4 |
| *Madagasikaria andersonii* | Ma | 569 | 16 |
| *Malpighia albiflora* | SA/CA | 3232.9 | 306.8 |
| *Malpighia cnide* | Ca | 1255.3 | 145 |
| *Malpighia coccigera* | Ca | 2257.1 | 367.7 |
| *Malpighia emarginata* | SA/Ca/CA/Me | 1163.8 | 93.7 |
| *Malpighia fucata* | Ca | 2285 | 324 |
| *Malpighia galeottiana* | Me | 546.7 | 15.9 |
| *Malpighia glabra* | SA/Ca/CA/Me | 1695.3 | 102.7 |
| *Malpighia incana* | Ca | 3817 | 270 |
| *Malpighia leticiana* | Me | 770.4 | 4.4 |
| *Malpighia lundellii* | CA/Me | 1453.3 | 127.3 |
| *Malpighia mexicana* | CA/Me | 852 | 18.9 |
| *Malpighia romeroana* | SA/CA/Me | 3250.1 | 324.6 |
| *Malpighia setosa* | Ca | NA | NA |
| *Malpighia souzae* | Me | 2834.2 | 193 |
| *Malpighia stevensii* | CA | 1389.9 | 14.8 |
| *Malpighia urens* | Ca | NA | NA |
| *Malpighia wendtii* | CA/Me | 2661 | 218.9 |
| *Malpighiodes bracteosa* | SA | 2194 | 271 |
| *Malpighiodes guianensis* | SA | 2626.1 | 335.8 |
| *Mascagnia affinis* | SA | 1423.6 | 68.3 |
| *Mascagnia almedae* | Me | NA | NA |
| *Mascagnia arenicola* | SA | 2698.2 | 310.8 |
| *Mascagnia australis* | SA | 1380.6 | 184.7 |
| *Mascagnia brevifolia* | SA | 731.2 | 40.3 |
| *Mascagnia cordifolia* | SA | 1685.3 | 78.1 |
| *Mascagnia dissimilis* | SA | 2821 | 438.3 |
| *Mascagnia divaricata* | SA/CA | 1860.7 | 229.7 |
| *Mascagnia eggersiana* | SA | 2194.2 | 217.8 |
| *Mascagnia lilacina* | Me | 509.5 | 42.6 |
| *Mascagnia polybotrya* | Me | 913.5 | 15.6 |
| *Mascagnia schunkei* | SA | 2768 | 591 |
| *Mascagnia strigulosa* | SA | 2300.8 | 340.4 |
| *Mascagnia tenuifolia* | SA | 2900 | 539 |
| *Mascagnia tomentosa* | CA/Me | 1710.9 | 20 |
| *Mascagnia vacciniifolia* | CA/Me | 3026.5 | 274.6 |
| *Mcvaughia bahiana* | SA | 564 | 63.7 |
| *Mezia angelica* | SA | 2421.7 | 259.3 |
| *Mezia araujoi* | SA | NA | NA |
| *Mezia beckii* | SA | 1756 | 118 |
| *Mezia includens* | SA/CA | 2897.8 | 390 |
| *Mezia mariposa* | SA | 2079.4 | 156 |
| *Mezia tomentosa* | SA | 3255 | 704 |
| *Microsteira ampihamensis* | Ma | NA | NA |
| *Microsteira argyrophylla* | Ma | 1537 | 5 |
| *Microsteira curtisii* | Ma | 1577 | 103 |
| *Microsteira diotostigma* | Ma | 1110 | 12.5 |
| *Microsteira paniculata* | Ma | NA | NA |
| *Mionandra camareoides* | SA | 512.2 | 11.4 |
| *Niedenzuella acutifolia* | SA | 1801.9 | 185.1 |
| *Niedenzuella sericea* | SA | 1772.8 | 258.5 |
| *Niedenzuella stannea* | SA/CA | 2349.4 | 261.9 |
| *Peixotoa bahiana* | SA | 963.5 | 3 |
| *Peixotoa cordistipula* | SA | 1477.7 | 69.4 |
| *Peixotoa glabra* | SA | 1440.1 | 28.3 |
| *Peixotoa hispidula* | SA | 1825.2 | 311.8 |
| *Peixotoa paludosa* | SA | 834.7 | 2.3 |
| *Peixotoa parviflora* | SA | 1365.5 | 122 |
| *Peixotoa reticulata* | SA | 1444.5 | 105.4 |
| *Peixotoa tomentosa* | SA | 1501.1 | 34.4 |
| *Philgamia glabrifolia* | Ma | 1404.7 | 46.4 |
| *Philgamia hibbertioides* | Ma | 1336.1 | 39.8 |
| *Psychopterys mcvaughii* | Me | 1345.5 | 23.5 |
| *Psychopterys rivularis* | CA/Me | 2563.1 | 177 |
| *Pterandra arborea* | SA | 2268.3 | 296 |
| *Ptilochaeta bahiensis* | SA | 749.2 | 71.8 |
| *Ptilochaeta nudipes* | SA | 1016 | 77 |
| *Rhynchophora humbertii* | Ma | 768 | 33.4 |
| *Rhynchophora phillipsonii* | Ma | 451.8 | 20.3 |
| *Spachea correae* | CA | 3968.6 | 384.1 |
| *Spachea elegans* | SA/Ca | 1785.2 | 183.9 |
| *Spachea membranacea* | SA/CA | 2540.5 | 147.5 |
| *Spachea tricarpa* | SA | 2824.8 | 542.5 |
| *Sphedamnocarpus angolensis* | Af | 1270 | 4 |
| *Sphedamnocarpus galphimiifolius* | Af | 752.4 | 31.6 |
| *Sphedamnocarpus poissoni* | Ma | NA | NA |
| *Sphedamnocarpus pruriens* | Af | 714.4 | 29.2 |
| *Stigmaphyllon aberrans* | SA | 1602 | 153.3 |
| *Stigmaphyllon bogotense* | SA/CA | 1893.5 | 236.9 |
| *Stigmaphyllon calcaratum* | SA | 1307.3 | 121.6 |
| *Stigmaphyllon ciliatum* | SA/Ca/CA | 1898.4 | 222.5 |
| *Stigmaphyllon cuzcanum* | SA | 1177.2 | 71.3 |
| *Stigmaphyllon ellipticum* | SA/CA/Me | 2101.2 | 113.6 |
| *Stigmaphyllon finlayanum* | SA | NA | NA |
| *Stigmaphyllon lindenianum* | SA/CA/Me | 2587.6 | 223 |
| *Stigmaphyllon paralias* | SA | 933.7 | 99.3 |
| *Stigmaphyllon pseudopuberum* | CA/Me | 2066.4 | 143.3 |
| *Stigmaphyllon puberum* | SA/Ca/CA | 3342.5 | 365.2 |
| *Stigmaphyllon retusum* | CA/Me | 1978.5 | 107.1 |
| *Stigmaphyllon sagraeanum* | Ca | 1283.5 | 90 |
| *Stigmaphyllon selerianum* | Me | 1114 | 30 |
| *Tetrapterys ambigua* | SA | 1399.3 | 101.2 |
| *Tetrapterys arcana* | CA/Me | 1794 | 130.3 |
| *Tetrapterys argentea* | CA/Me | 2378.3 | 188.7 |
| *Tetrapterys cardiophylla* | SA | 919.3 | 64.8 |
| *Tetrapterys discolor* | SA/CA/Me | 2606.6 | 221.7 |
| *Tetrapterys fimbripetala* | SA | 2535 | 297.2 |
| *Tetrapterys goudotiana* | SA/CA | 2731.1 | 123.1 |
| *Tetrapterys heterophylla* | CA/Me | 1094.1 | 28.7 |
| *Tetrapterys maranhamensis* | SA | 2520.5 | 169.7 |
| *Tetrapterys megalantha* | SA | 2660.8 | 293.3 |
| *Tetrapterys mexicana* | Me | 1035.6 | 21.5 |
| *Tetrapterys microphylla* | SA | 1434.3 | 29 |
| *Tetrapterys mucronata* | SA/CA | 2386.3 | 276.2 |
| *Tetrapterys paludosa* | SA | 737.3 | 61.3 |
| *Tetrapterys phlomoides* | SA | 1495.8 | 153.2 |
| *Tetrapterys pusilla* | SA | 1846 | 174 |
| *Tetrapterys salicifolia* | SA | 1391 | 193 |
| *Tetrapterys schiedeana* | CA/Me | 1963.9 | 129.5 |
| *Tetrapterys seleriana* | CA/Me | 1259 | 101.6 |
| *Tetrapterys styloptera* | SA/CA | 2413.9 | 247.5 |
| *Tetrapterys tinifolia* | SA/CA/Me | 3142 | 296.5 |
| *Tetrapterys vacciniifolia* | SA | NA | NA |
| *Thryallis laburnum* | SA | 1088.5 | 95 |
| *Thryallis latifolia* | SA | 1355 | 42 |
| *Thryallis longifolia* | SA | 810.6 | 63 |
| *Thryallis parviflora* | SA | 1420.5 | 21 |
| *Triaspis glaucophylla* | Af | 703.1 | 22.3 |
| *Triaspis hypericoides* | Af | 441.3 | 13.8 |
| *Triaspis nelsonii* | Af | 599.7 | 14.7 |
| *Triaspis niedenzuiana* | Af | 535.5 | 29 |
| *Triaspis odorata* | Af | 1135.8 | 78.5 |
| *Tricomaria usillo* | SA | 231.4 | 8.6 |
| *Triopterys jamaicensis* | Ca | 927 | 93 |
| *Triopterys paniculata* | Ca | NA | NA |
| *Tristellateia africana* | Af | 959.5 | 66 |
| *Tristellateia australasiae* | As | 2407.6 | 203.9 |
| *Tristellateia madagascariensis* | Ma | 1766.5 | 167.4 |
| *Verrucularia glaucophylla* | SA | 830.2 | 57 |

**Table S3.** Species sampling of biomes by genera. For each genus, the expected number of species among each biome was estimated using geo-referenced data from GBIF and presence/absence across five main biomes as designated by the World Wildlife Fund http://www.worldwildlife.org/science/wildfinder/; Grass, grassland/savannah; MBTF, moist broadleaf tropical forest; Xeric, desert/Mediterranean scrub; SDTF, seasonally dry tropical forest; TCF, tropical coniferous forest). The notation below designates ‘number of species from GBIF database/number of species sampled in our phylogeny’. These expectations were compared to the sampling distribution of species included in our phylogeny using a χ^2^-test. Significant deviations from the GBIF expectation are indicated by a P-value < 0.05 and in bold.

|  | GBIF Sample (n = 828) | | | | | Phylo. Sample (n = 357) | | | | |  |
| --- | --- | --- | --- | --- | --- | --- | --- | --- | --- | --- | --- |
| **Genus** | **Grass** | **MBTF** | **Xeric** | **SDTF** | **TCF** | **Grass** | **MBTF** | **Xeric** | **SDTF** | **TCF** | **P-value** |
| *Acmanthera* |  | 2 |  |  |  |  | 1 |  |  |  | 0.9735 |
| *Acridocarpus* | 6 | 17 | 2 | 2 |  | 4 | 4 | 1 | 2 |  | 0.0254 |
| *Adelphia* |  | 2 |  |  |  |  | 2 |  |  |  | 1.0000 |
| *Aenigmatanthera* |  |  |  | 1 |  |  |  |  | 1 |  | 1.0000 |
| *Alicia* | 1 | 1 |  |  |  | 1 | 1 |  |  |  | 1.0000 |
| *Amorimia* |  | 2 |  | 3 |  |  | 1 |  | 3 |  | 0.9735 |
| *Aspicarpa* | 2 | 1 | 3 | 2 |  | 1 | 1 | 3 | 3 |  | 0.9098 |
| *Aspidopterys* |  | 2 |  |  | 1 |  | 1 |  |  |  | 0.9735 |
| *Banisteriopsis* | 24 | 24 | 1 | 4 |  | 13 | 13 | 1 | 2 |  | **0.0256** |
| *Barnebya* |  |  | 1 |  |  |  |  |  |  |  | NA |
| *Blepharandra* | 1 | 4 |  |  |  | 1 | 2 |  |  |  | 0.9098 |
| *Brachylophon* | 1 |  |  |  |  |  |  |  |  |  | NA |
| *Bronwenia* | 2 | 5 |  | 2 |  |  | 4 |  | 1 |  | 0.9513 |
| *Bunchosia* | 1 | 36 |  | 16 | 2 | 1 | 18 |  | 10 | 2 | **0.0239** |
| *Burdachia* | 1 | 4 |  |  |  |  | 1 |  |  |  | 0.6899 |
| *Byrsonima* | 29 | 72 | 5 | 3 |  | 3 | 4 | 2 |  |  | **0.0000** |
| *Calcicola* |  |  |  | 2 |  |  |  | 2 |  |  | 1.0000 |
| *Callaeum* |  | 4 | 1 | 2 | 2 |  | 3 | 1 | 2 | 1 | 0.9450 |
| *Camarea* | 4 |  | 1 |  |  | 1 |  | 1 |  |  | 0.6899 |
| *Carolus* |  | 2 |  | 2 |  |  | 2 |  | 2 |  | 1.0000 |
| *Caucanthus* | 2 |  |  |  |  | 1 |  |  |  |  | 0.9735 |
| *Christianella* |  | 4 |  | 1 |  |  | 2 |  | 1 |  | 0.9098 |
| *Coleostachys* |  | 1 |  |  |  |  | 1 |  |  |  | 1.0000 |
| *Cordobia* |  |  |  | 1 |  |  |  |  | 1 |  | 1.0000 |
| *Cottsia* |  |  | 3 |  |  |  |  | 2 |  |  | 0.9876 |
| *Diacidia* |  | 8 |  |  |  |  | 2 |  |  |  | 0.3425 |
| *Diaspis* | 1 |  |  |  |  | 1 |  |  |  |  | 1.0000 |
| *Dicella* |  | 5 |  | 1 |  |  | 3 |  | 2 |  | 0.7725 |
| *Digoniopterys* |  |  | 1 |  |  |  |  | 1 |  |  | 1.0000 |
| *Dinemagonum* |  |  | 1 |  |  |  |  | 1 |  |  | 1.0000 |
| *Dinemandra* |  |  | 1 |  |  |  |  | 1 |  |  | 1.0000 |
| *Diplopterys* | 5 | 18 | 1 | 1 |  | 3 | 5 |  | 3 |  | **0.0067** |
| *Echinopterys* |  |  |  | 2 |  |  |  | 2 |  |  | 1.0000 |
| *Ectopopterys* |  | 1 |  |  |  |  | 1 |  |  |  | 1.0000 |
| *Excentradenia* |  | 2 |  |  |  |  | 1 |  |  |  | 0.9735 |
| *Flabellaria* | 1 |  |  |  |  | 1 |  |  |  |  | 1.0000 |
| *Flabellariopsis* |  | 1 |  |  |  |  | 1 |  |  |  | 1.0000 |
| *Gallardoa* | 1 |  |  |  |  | 1 |  |  |  |  | 1.0000 |
| *Galphimia* | 4 | 2 | 1 | 10 | 8 | 2 | 2 | 1 | 6 | 2 | 0.1307 |
| *Gaudichaudia* |  | 1 |  | 6 | 3 |  | 1 |  | 6 | 1 | 0.8557 |
| *Glandonia* |  | 3 |  |  |  |  | 1 |  |  |  | 0.8557 |
| *Heladena* | 1 |  |  |  |  | 1 |  |  |  |  | 1.0000 |
| *Henleophytum* |  |  |  |  |  |  |  |  |  |  | NA |
| *Heteropterys* | 27 | 64 | 7 | 12 |  | 5 | 19 | 2 | 4 |  | **0.0000** |
| *Hiptage* |  | 3 |  |  |  |  | 1 |  |  |  | 0.8557 |
| *Hiraea* | 1 | 32 |  | 2 |  |  | 3 |  | 1 |  | **0.0000** |
| *Janusia* | 1 | 5 | 2 |  |  | 1 | 4 | 2 |  |  | 0.9953 |
| *Jubelina* |  | 6 |  |  |  |  | 4 |  |  |  | 0.9554 |
| *Lasiocarpus* |  |  |  | 2 |  |  |  |  |  |  | NA |
| *Lophanthera* |  | 4 |  |  |  |  | 4 |  |  |  | 1.0000 |
| *Lophopterys* |  | 5 |  |  |  |  | 2 |  |  |  | 0.7725 |
| *Madagasikaria* |  |  | 1 |  |  |  |  | 1 |  |  | 1.0000 |
| *Malpighia* | 1 | 15 | 1 | 12 | 2 |  | 9 | 1 | 5 |  | 0.1658 |
| *Malpighiodes* |  | 3 |  |  |  |  | 2 |  |  |  | 0.9876 |
| *Mascagnia* | 2 | 18 | 1 | 5 |  | 2 | 8 | 1 | 4 |  | 0.2182 |
| *Mcvaughia* |  |  | 1 |  |  |  |  | 1 |  |  | 1.0000 |
| *Mezia* |  | 8 |  |  |  |  | 5 |  |  |  | 0.8903 |
| *Microsteira* |  | 4 |  | 3 |  |  | 1 |  | 3 |  | 0.6899 |
| *Mionandra* |  |  |  | 1 |  |  |  |  | 1 |  | 1.0000 |
| *Niedenzuella* | 2 | 9 | 1 |  |  | 1 | 2 |  |  |  | 0.2033 |
| *Peixotoa* | 14 | 4 |  | 3 |  | 4 | 1 |  | 3 |  | 0.0520 |
| *Philgamia* |  | 4 |  |  |  |  |  |  | 2 |  | 1.0000 |
| *Psychopterys* |  | 4 |  | 3 | 1 |  | 1 |  | 1 | 1 | 0.4653 |
| *Pterandra* | 1 | 9 |  |  |  |  | 1 |  |  |  | 0.1301 |
| *Ptilochaeta* |  | 1 | 2 | 1 |  |  |  | 1 | 1 |  | 0.9735 |
| *Rhynchophora* |  |  | 1 | 1 |  |  |  | 1 | 1 |  | 1.0000 |
| *Spachea* |  | 5 |  |  |  |  | 4 |  |  |  | 0.9953 |
| *Sphedamnocarpus* | 4 | 1 |  |  |  | 3 |  |  |  |  | 0.9928 |
| *Stigmaphyllon* | 2 | 6 | 3 | 7 |  | 2 | 8 | 1 | 2 |  | 0.2335 |
| *Tetrapterys* | 8 | 35 | 2 | 5 | 1 | 4 | 13 | 2 | 2 |  | **0.0015** |
| *Thryallis* | 3 |  | 1 | 1 |  | 2 |  | 1 | 1 |  | 0.9876 |
| *Triaspis* | 7 | 4 | 1 |  |  | 5 |  | 1 |  |  | 0.9662 |
| *Tricomaria* |  |  | 1 |  |  |  |  | 1 |  |  | 1.0000 |
| *Triopterys* |  | 1 |  | 1 |  |  | 1 |  |  |  | 1.0000 |
| *Tristellateia* |  | 3 | 1 | 1 |  | 1 | 2 |  |  |  | 0.9876 |
| *Verrucularia* |  | 1 | 1 |  |  |  |  | 1 |  |  | 1.0000 |

**Table S4.** Species sampling of New World geographic regions by genera. For each genus with <50% taxon sampling, the expected number of species among each region was estimated based on geo-referenced data from GBIF and presence/absence data from the four main geographic regions used in our analyses (SA, South America; CA, Central America; Ca, Carribbean; and Me, Mexico). The notation below designates ‘number of species from GBIF database/number of species sampled in our phylogeny’. These expectations were compared to the sampling distribution of species included in our phylogeny using a χ^2^-test. Significant deviations from the GBIF expectation are indicated by a P-value < 0.05 and in bold.

| **Genus** | **SA** | **CA** | **Ca** | **Me** | **SA/CA** | **SA/Me** | **SA/Ca** | **CA/Me** | **Ca/Me** | **SA/CA/Me** | **SA/CA/Ca/Me** | **P-value** |
| --- | --- | --- | --- | --- | --- | --- | --- | --- | --- | --- | --- | --- |
| *Pterandra* | 8/1 | 2/0 |  |  |  |  |  |  |  |  |  | 0.8649 |
| *Byrsonima* | 95/7 | 2/0 | 4/1 |  | 5/0 |  |  | 1/0 |  | 1/0 | 1/1 | **0.0000** |
| *Hiraea* | 25/1 | 2/0 |  |  | 2/0 |  |  | 2/1 |  | 4/3 |  | **0.0136** |
| *Acmanthera* | 2/1 |  |  |  |  |  |  |  |  |  |  | 1.0000 |
| *Stigmaphyllon* | 50/5 | 4/0 | 4/1 | 1/1 | 5/1 |  | 1/0 | 2/2 | 1/0 | 3/2 | 1/0 | **0.0000** |
| *Niedenzuella* | 10/2 |  |  |  | 1/1 | 1/0 |  |  |  |  |  | 0.8454 |
| *Diacidia* | 8/2 |  |  |  |  |  |  |  |  |  |  | 0.9529 |
| *Aspicarpa* | 5/5 |  |  | 3/3 |  |  |  |  |  |  |  | 1.0000 |
| *Burdachia* | 5/1 |  |  |  |  |  |  |  |  |  |  | 0.9878 |
| *Heteropterys* | 94/31 | 6/0 | 2/0 | 2/0 | 1/0 | 1/0 |  | 1/2 |  | 2/2 | 1/1 | **0.0000** |
| *Camarea* | 0/2 |  |  |  |  |  |  |  |  |  |  |  |
| *Excentradenia* | 2/1 |  |  |  |  |  |  |  |  |  |  | 1.0000 |
| *Psychopterys* |  | 2/0 |  | 4/2 |  |  |  | 2/1 |  |  |  | 0.9996 |
| *Peixotoa* | 21/8 |  |  |  |  |  |  |  |  |  |  | 0.7090 |
| *Lophopterys* | 5/2 |  |  |  |  |  |  |  |  |  |  | 0.9991 |
| *Tetrapterys* | 34/11 | 3/0 | 1/0 | 1/1 | 4/3 |  |  | 5/5 |  | 3/2 |  | 0.1359 |
| *Glandonia* | 3/1 |  |  |  |  |  |  |  |  |  |  | 0.9998 |
| *Diplopterys* | 22/10 |  |  | 1/0 | 2/1 |  |  |  |  |  |  | 0.7954 |
| *Malpighia* |  | 1/1 | 7/6 | 12/3 | 3/1 |  |  | 5/3 |  | 0/1 | 2/2 | 0.6195 |
| *Lophanthera* | 3/3 | 1/1 |  |  |  |  |  |  |  |  |  | 1.0000 |
| *Gaudichaudia* |  | 1/1 |  | 8/7 |  |  |  | 1/0 |  | 0/1 |  | 1.0000 |
| *Mascagnia* | 19/10 |  |  | 2/3 | 2/1 |  |  | 2/2 |  | 1/0 |  | 0.9178 |

**Table S5.** Species sampling bias of SDTF across New World geographic regions. Regions include South America (SA), Central America (CA), and Mexico (Me). The expected number of species in each region is based on geo-referenced data from GBIF and presence/absence data in a given region. The notation below designates ‘number of species from GBIF database/number of species sampled in our phylogeny’. The regional affiliations of the species present in our phylogeny derived from Anderson et al. (2006) and Anderson (personal communication). The overall expected GBIF distribution was compared to the sampling distribution present in our phylogeny using a χ^2^-test. Our sampling was significantly different then that expectation (P-value = 0.0293).

| **Region** | **SDTF GBIF Sample** | **SDTF Phylo. Sample** |
| --- | --- | --- |
| CA | 7 | 1 |
| CA/Me | 6 | 7 |
| Car | 7 | 3 |
| Car/Me | 2 |  |
| Me | 38 | 22 |
| SA | 44 | 33 |
| SA/CA | 1 |  |
| SA/CA/Car/Me | 1 | 1 |
| SA/CA/Me | 1 | 1 |
|  |  |  |

**Table S6.** Evolutionary lag times accounting for taxon sampling biases. Lag times represent the mean time (Myr) from when a lineage became geographically restricted to Mexico and when it evolve a given climate threshold. Positive values indicate post-adaptation, negative values indicate pre-adaptation. The original means were calculated based on all taxa, revised means were calculated excluded lineages with known sampling biases (*Bunchosia*, *Stigmaphyllon*, and *Tetrapterys*). Q indicated the 95% quantile range of the mean.

|  |  | **Revised** | | | **Original** | | |
| --- | --- | --- | --- | --- | --- | --- | --- |
| **Variable** | **Threshold** | **mean** | **Q2.5** | **Q97.5** | **mean** | **Q2.5** | **Q97.5** |
| *Total Annual Precipitation* | 1800mm | -29.68 | -34.94 | -22.78 | -22.00 | -36.00 | -21.60 |
|  | 1600mm | -19.37 | -26.48 | -14.17 | -15.80 | -23.30 | -11.60 |
| *Precipitation During Driest Quarter* | 100m | -3.60 | -8.14 | 0.25 | -3.23 | -6.75 | -0.40 |
|  | 50mm | 1.38 | 0.20 | 3.30 | 0.71 | -0.22 | 2.16 |

**Table S7.** Biogeographic model comparison. Models compared included: the “dispersal-extinction-cladogenesis” model (DEC), the “dispersal-vicariance analysis” model (DIVA), and the “Bayesian inference of historical biogeography for discrete areas” model (BAYAREA). We modified these models to account for founder-event speciation events (+J in DEC+J). Maximum likelihood best-fit estimates are included for dispersal probability (d), extinction probability (e), and founder‐event speciation probability (j) parameters. Models were compared using a likelihood ratio test based on a χ^2^-test of the D statistic calculated relative to the best-fit model (DEC+J).

| **Model** | **d** | **e** | **j** | **LnL** | **AIC** | **D** | **Parameters** | **P** |
| --- | --- | --- | --- | --- | --- | --- | --- | --- |
| DEC+J | 0.0112 | 0.0000 | 0.0140 | -605.2 | 1216 | 0.0 | 3 | - |
| DEC | 0.0129 | 0.0005 | - | -618.5 | 1241 | -26.6 | 2 | < 0.000 |
| DIVA+J | 0.0127 | 0.0000 | 0.0140 | -628.4 | 1263 | -46.4 | 3 | < 0.000 |
| BAYAREA+J | 0.0082 | 0.0000 | 0.0334 | -628.4 | 1263 | -46.4 | 3 | < 0.000 |
| DIVA | 0.0151 | 0.0000 | - | -645.4 | 1295 | -80.4 | 2 | < 0.000 |
| BAYAREA | 0.0108 | 0.0249 | - | -699.7 | 1403 | -189.0 | 2 | < 0.000 |

**Table S8.** Comparison of precipitation variables across Mexican (MM) and Non-Mexican Malpighiaceae using phylogenetic generalized linear models (PGLM). PGLM corrects for phylogenetic signal (Pagel’s λ) in the dependent variable (here: total annual precipitation and precipitation seasonality). Β, regression estimate; SE, standard error of regression estimate. t, t-statistic for computing significance. P-values indicated significant differences between MM and non-MM. Values represent mean values estimated across 100 ML trees.

|  |  | Total Annual Precipitation (mm) | Precipitation Seasonality (mm) |
| --- | --- | --- | --- |
| Phylogenetic signal | λ | 0.67 | 0.68 |
| Non-MM | β | 2038.6 | 195.8 |
|  | SE | 329.7 | 49.5 |
| MM, endemic | β | 1571.4 | 49.2 |
|  | SE | 364.4 | 21.0 |
|  | t | -1.3 | -7.0 |
|  | P | 0.2200 | < 0.001 |
| MM, widspread | β | 2223.6 | 195.0 |
|  | SE | 537.0 | 20.6 |
|  | t | 0.3 | -0.0 |
|  | P | 0.7300 | 0.9300 |

**Table S9 –** Incomplete taxon sampling proportions used for diversification analysis using BAMM. Species numbers (N) designates ‘total number of species/number of species sampled in our phylogeny’. Total number of species is based on Anderson et al. (2006 and onward).

| **Genus** | **N** | **Sampling Fraction** |
| --- | --- | --- |
| *Diaspis* | 1/0 | 0.000 |
| *Lasiocarpus* | 2/0 | 0.000 |
| *Cottsia* | 3/0 | 0.000 |
| *Pterandra* | 15/1 | 0.067 |
| *Byrsonima* | 129/9 | 0.070 |
| *Aspidopterys* | 11/1 | 0.091 |
| *Hiraea* | 51/5 | 0.098 |
| *Acmanthera* | 7/1 | 0.143 |
| *Stigmaphyllon* | 93/14 | 0.151 |
| *Niedenzuella* | 18/3 | 0.167 |
| *Diacidia* | 11/2 | 0.182 |
| *Aspicarpa* | 41/8 | 0.195 |
| *Burdachia* | 5/1 | 0.200 |
| *Heteropterys* | 143/34 | 0.238 |
| *Excentradenia* | 4/1 | 0.250 |
| *Camarea* | 8/2 | 0.250 |
| *Psychopterys* | 8/2 | 0.250 |
| *Peixotoa* | 29/8 | 0.276 |
| *Lophopterys* | 7/2 | 0.286 |
| *Tetrapterys* | 69/22 | 0.319 |
| *Glandonia* | 3/1 | 0.333 |
| *Diplopterys* | 31/11 | 0.355 |
| *Malpighia* | 47/17 | 0.362 |
| *Lophanthera* | 11/4 | 0.364 |
| *Tristellateia* | 8/3 | 0.375 |
| *Gaudichaudia* | 21/9 | 0.429 |
| *Acridocarpus* | 27/13 | 0.481 |
| *Mascagnia* | 37/18 | 0.486 |
| *Triopterys* | NA/0 | 0.486 |
| *Banisteriopsis* | 65/32 | 0.492 |
| *Barnebya* | 2/1 | 0.500 |
| *Caucanthus* | 2/1 | 0.500 |
| *Mionandra* | 2/1 | 0.500 |
| *Verrucularia* | 2/1 | 0.500 |
| *Adelphia* | 4/2 | 0.500 |
| *Malpighiodes* | 4/2 | 0.500 |
| *Philgamia* | 4/2 | 0.500 |
| *Ptilochaeta* | 4/2 | 0.500 |
| *Blepharandra* | 6/3 | 0.500 |
| *Triaspis* | 10/5 | 0.500 |
| *Bunchosia* | 68/35 | 0.515 |
| *Galphimia* | 26/14 | 0.538 |
| *Sphedamnocarpus* | 7/4 | 0.571 |
| *Christianella* | 5/3 | 0.600 |
| *Mezia* | 10/6 | 0.600 |
| *Dicella* | 8/5 | 0.625 |
| *Microsteira* | 8/5 | 0.625 |
| *Janusia* | 14/9 | 0.643 |
| *Rhynchophora* | 3/2 | 0.667 |
| *Carolus* | 6/4 | 0.667 |
| *Jubelina* | 6/4 | 0.667 |
| *Spachea* | 6/4 | 0.667 |
| *Amorimia* | 10/7 | 0.700 |
| *Bronwenia* | 10/7 | 0.700 |
| *Callaeum* | 11/8 | 0.727 |
| *Thryallis* | 5/4 | 0.800 |
| *Brachylophon* | 1/1 | 1.000 |
| *Coleostachys* | 1/1 | 1.000 |
| *Cordobia* | 1/1 | 1.000 |
| *Digoniopterys* | 1/1 | 1.000 |
| *Dinemagonum* | 1/1 | 1.000 |
| *Dinemandra* | 1/1 | 1.000 |
| *Ectopopterys* | 1/1 | 1.000 |
| *Flabellaria* | 1/1 | 1.000 |
| *Flabellariopsis* | 1/1 | 1.000 |
| *Gallardoa* | 1/1 | 1.000 |
| *Henleophytum* | 1/1 | 1.000 |
| *Madagasikaria* | 1/1 | 1.000 |
| *Mcvaughia* | 1/1 | 1.000 |
| *Tricomaria* | 1/1 | 1.000 |
| *Aenigmatanthera* | 2/2 | 1.000 |
| *Alicia* | 2/2 | 1.000 |
| *Calcicola* | 2/2 | 1.000 |
| *Echinopterys* | 2/2 | 1.000 |
| *Hiptage* | 6/6 | 1.000 |
| *Heladena* | 1/2 | 1.000 |

**References:**

Anderson, W. R., Anderson, C., and Davis, C. C. (2006). http://herbarium.lsa.umich.edu/malpigh/index.html. *Malpighiaceae*.

Pennington, R. T., Lavin, M., and Oliveira-Filho, A. (2009). Woody Plant Diversity, Evolution, and Ecology in the Tropics: Perspectives from Seasonally Dry Tropical Forests. *Annu. Rev. Ecol. Evol. Syst.* 40, 437–457.

**Figure S1.** Map of geographic regions where Malpighiaceae occur. Colors of geographic areas correspond to those shown in Figs 3 and 4 of the main text. Red line indicates approximate latitudinal range limits of Malpighiaceae (36° N, 37° S).

**Figure S2.** Phylogeny and divergence time estimates of Malpighiaceae. Phylogeny based on best-fit ML tree with branch lengths inferred using BEAST. The three fossil calibration points described in the main text are labeled using asterisks. The biogeograhpic distribution for each Malpighiaceae species is shown in parentheses  (SA, South America; CA, Central America; Me, Mexico; Ca, Caribbean; As, Asia; Af, Africa; and M, Madagascar). Species that are endemic to Mexico are shown in green. Species that are distributed across Mexico plus other New World regions are shown in red. Numbers above branches are Bayesian posterior probabilities/maximum likelihood bootstrap percentages. Divergence time estimates in million years with associated confidence intervals shown in blue.

**Figure S3.** Age and geographic origins of Mexican endemics. Mean date of each geographic restriction to Mexico estimated from Lagrange for stem, parent node. Error bars represent 95% confidence intervals estimated across mean ages for 100 ML bootstrap trees. Plot ordered by age of stem node from oldest to youngest. Pie-charts represent the estimated proportion of the ancestral range at the parental node, which represents each lineage’s range prior to its restriction to Mexico. Key in upper right indicates ancestral range reconstructions. E, Eocene; P, Pliocene; Q, Quaternary (Pleistocene/Holocene).

**Figure S4.** Evolutionary lag times of total annual precipitation. Mean lag times represent the time between when a lineage became geographically restricted to Mexico and when the lineage adapted to a given threshold for total annual precipitation. These thresholds are consistent with modern seasonally dry tropical forest in Mexico (≤1,800 mm yr-1, blue; Pennington et al., 2009), and drier conditions inhabited by Malpighiaceae in Mexico (≤1,600 mm yr^-1^, light blue, see Fig. 2). Negative lag times indicate pre-adaptation, i.e., the lineage adapted to the threshold before becoming restricted to Mexico. Positive lag times indicate *in situ* adaptation, i.e., the lineage adapted to the threshold after becoming restricted to Mexico. Pie-charts represent the estimated ancestral range at the stem, parent node of each lineage. Parent node range represents the original range, prior to adaptation to the given precipitation threshold. Key in upper right indicates ancestral range reconstructions for stem, parent nodes. Error bars represent 95% confidence intervals estimated across mean ages from 100 ML bootstrap trees.

**Figure S5.** Evolutionary lag times of precipitation seasonality. Mean lag times represent the time between when a lineage became geographically restricted to Mexico and when the lineage adapted to a given threshold for precipitation seasonality. These thresholds include those consistent with modern seasonally dry tropical forest in Mexico (≤50 mm qtr^-1^, red; Pennington et al., 2009), and more moderate historic levels (≤100 mm qtr^-1^, yellow). Negative lag times indicate pre-adaptation, i.e., the lineage adapted to the threshold before becoming restricted to Mexico. Positive lag times indicate *in situ* adaptation, i.e., the lineage adapted to the threshold after becoming restricted to Mexico. Pie-charts represent the estimated ancestral range at the stem, parent node of each lineage. Parent node range represents the original range, prior to adaptation to the given precipitation threshold. Key in upper right indicates ancestral range reconstructions for stem, parent nodes. Error bars represent 95% confidence intervals estimated across mean ages from 100 ML bootstrap trees.

**Figure S6.** Major shifts in diversification rate through time in Malpighiceae. Phylogeny is the time-calibrated best-fit ML tree (Fig. S2). Branches are colored by mean marginal density estimates of net diversification rates (see key for corresponding shading of rate estimates). Nodes with a significant shift in diversification rate, based on the cumulative shift probability ≥ 0.5, are indicated with a black dot.

**Figure S7.** Frequency distribution of biome types for Mexican and non-Mexican Malpighiaceae. Mexican Malpighiaceae are further divided into two groups: lineages that are geographically restricted to Mexico, and wide-ranging lineages that also occur outside of Mexico. Biome occurrence data is based on pairing species geo-referenced data with World Wildlife Fund biome designations (http://www.worldwildlife.org/science/wildfinder/).
