# Supplementary figures and images for: The establishment of Central American migratory corridors and the biogeographic origins of seasonally dry tropical forests in Mexico
Source: Front Genet. 2014 Dec 19;5:433. doi: 10.3389/fgene.2014.00433 (PMC4271706; doi:10.3389/fgene.2014.00433)

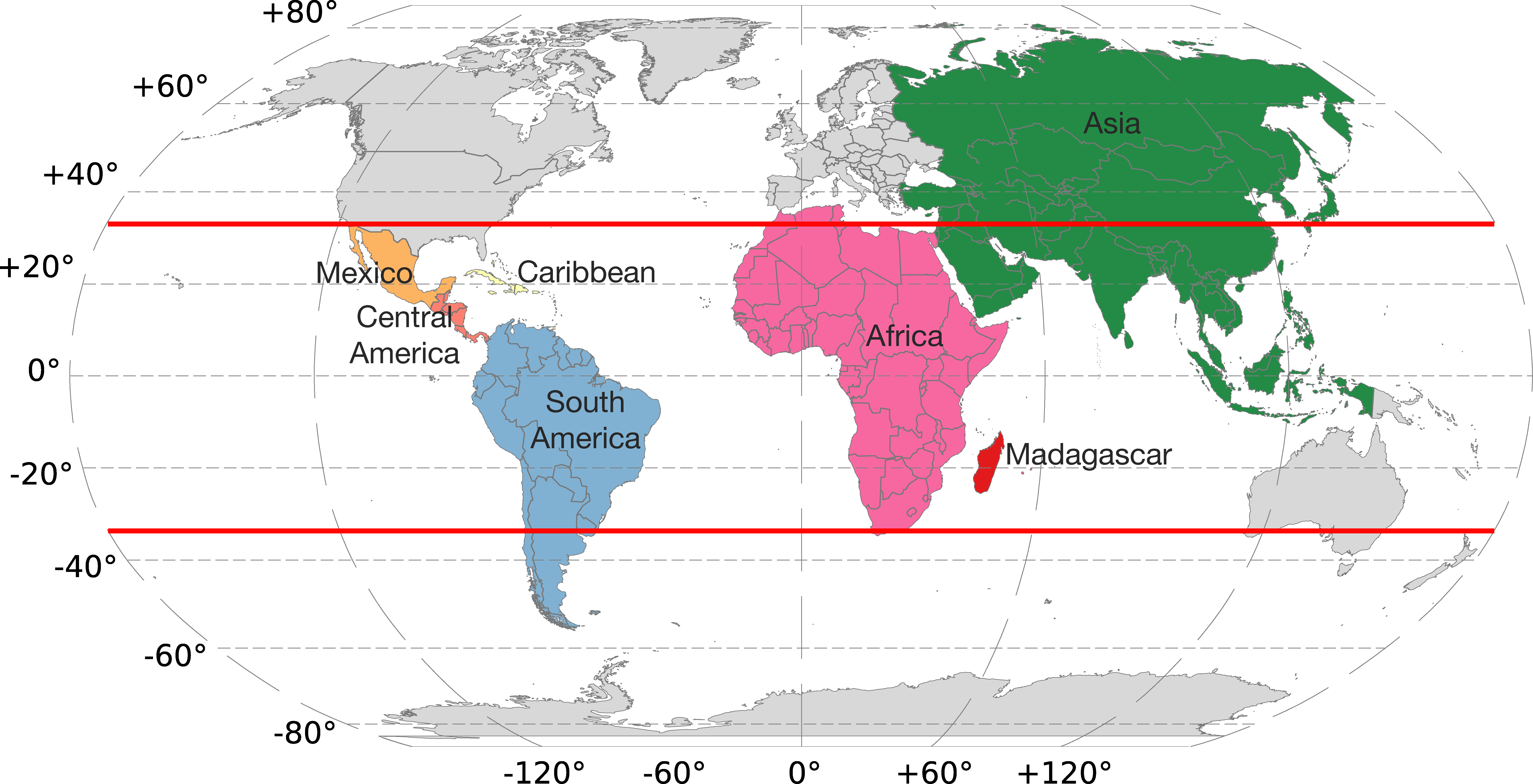

Supplement: Supplementary file 2 [file Image1.TIF]

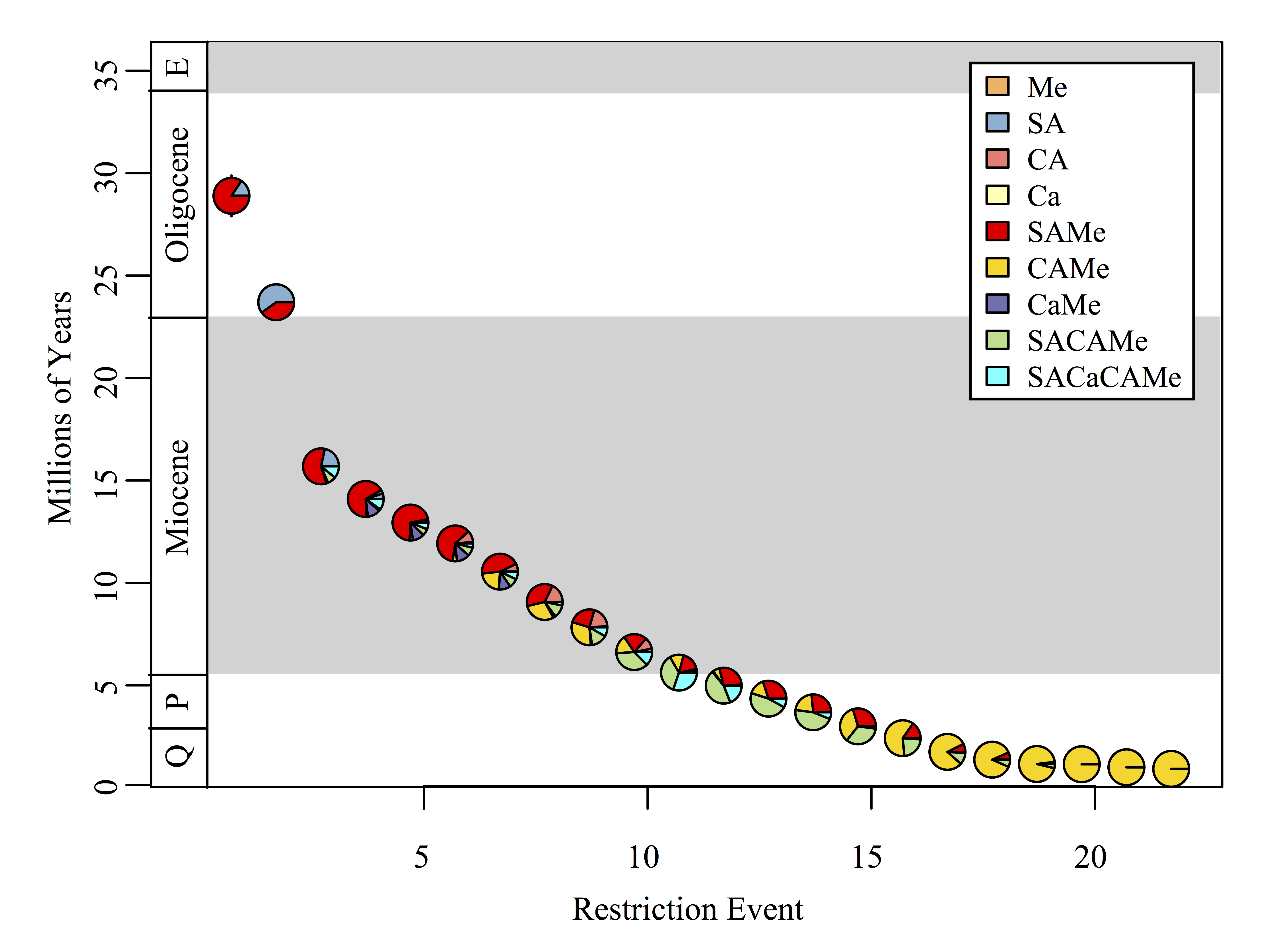

Supplement: Supplementary file 4 [file Image3.TIF]

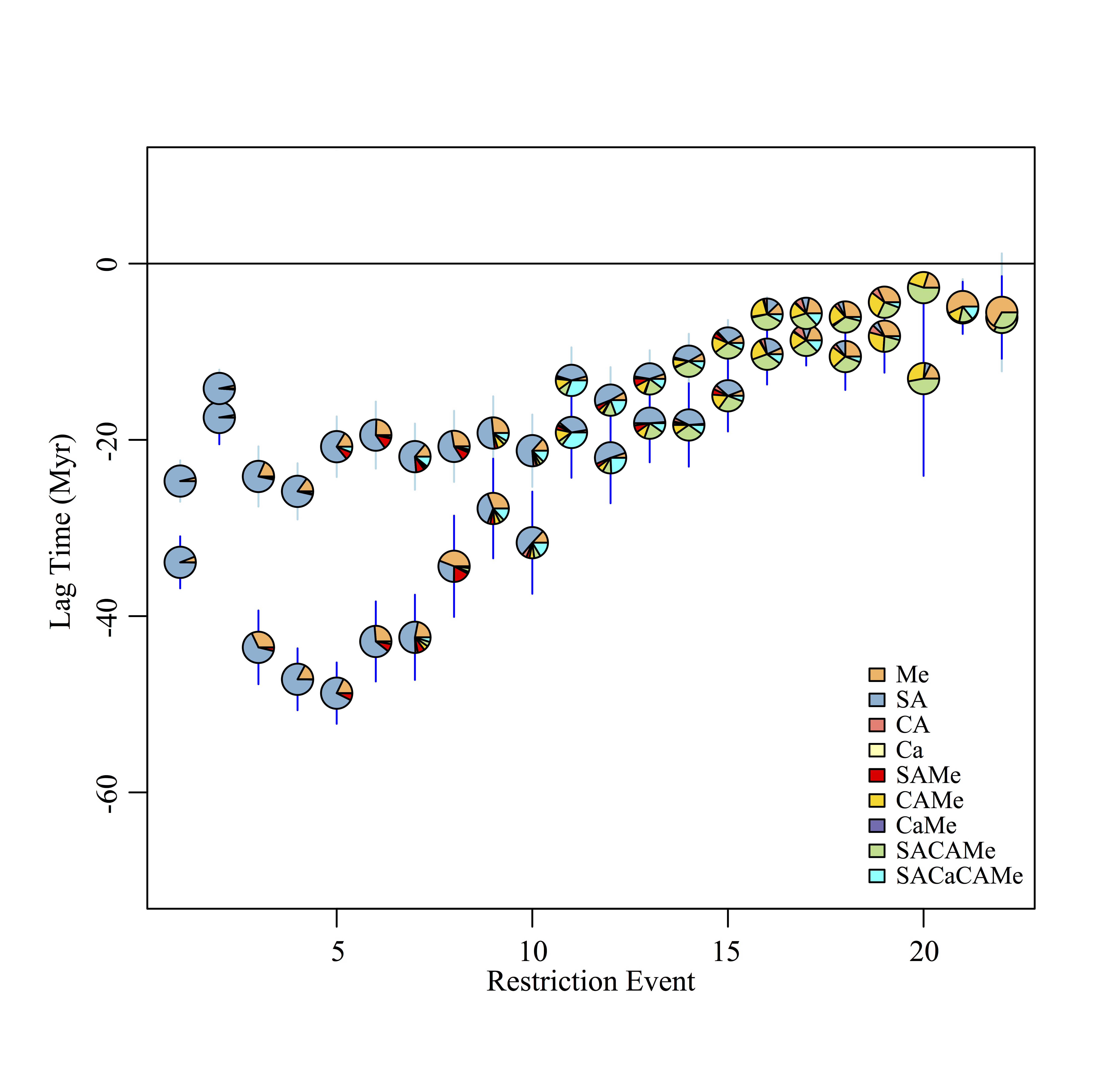

Supplement: Supplementary file 5 [file Image4.TIF]

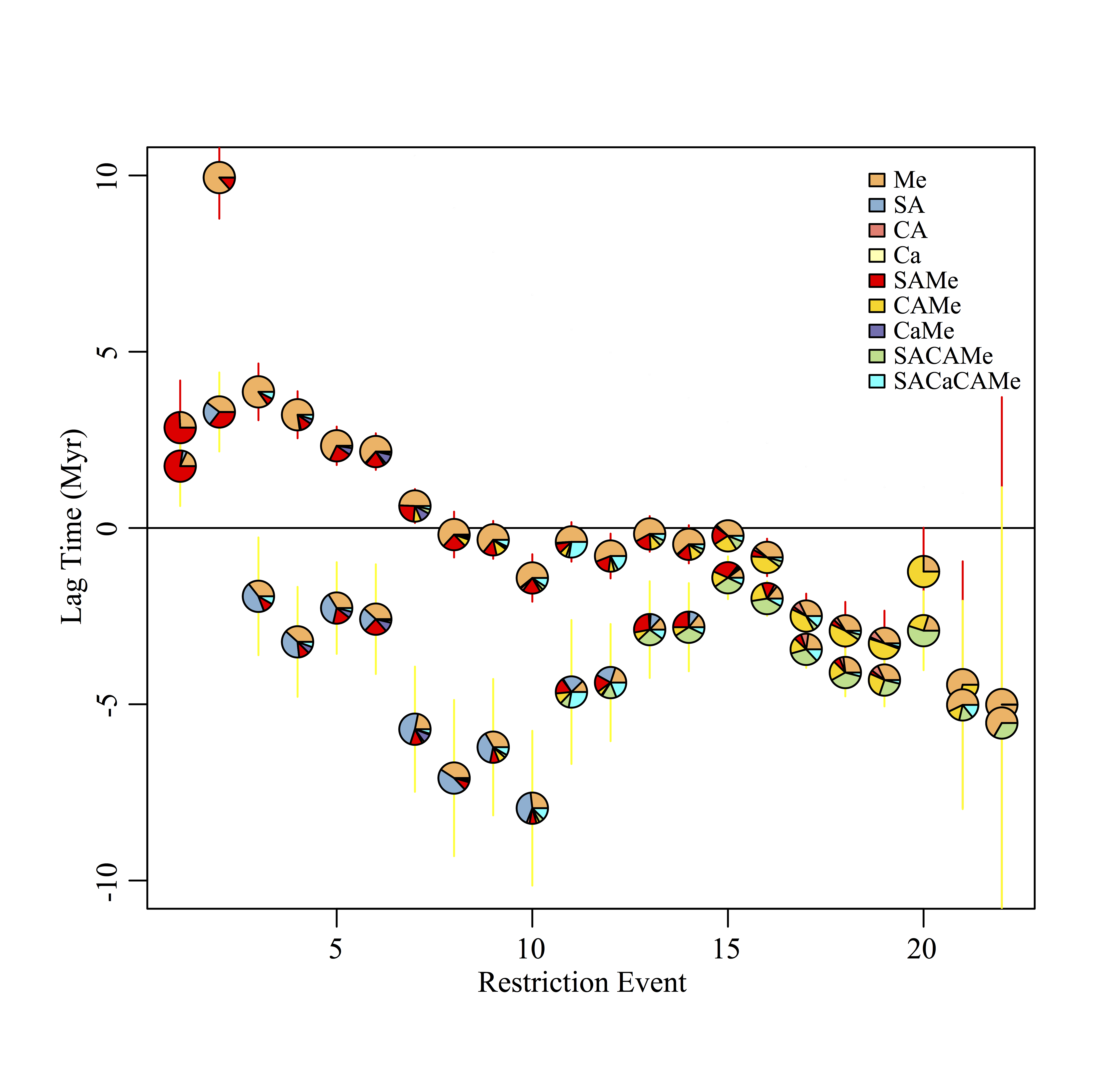

Supplement: Supplementary file 6 [file Image5.TIF]

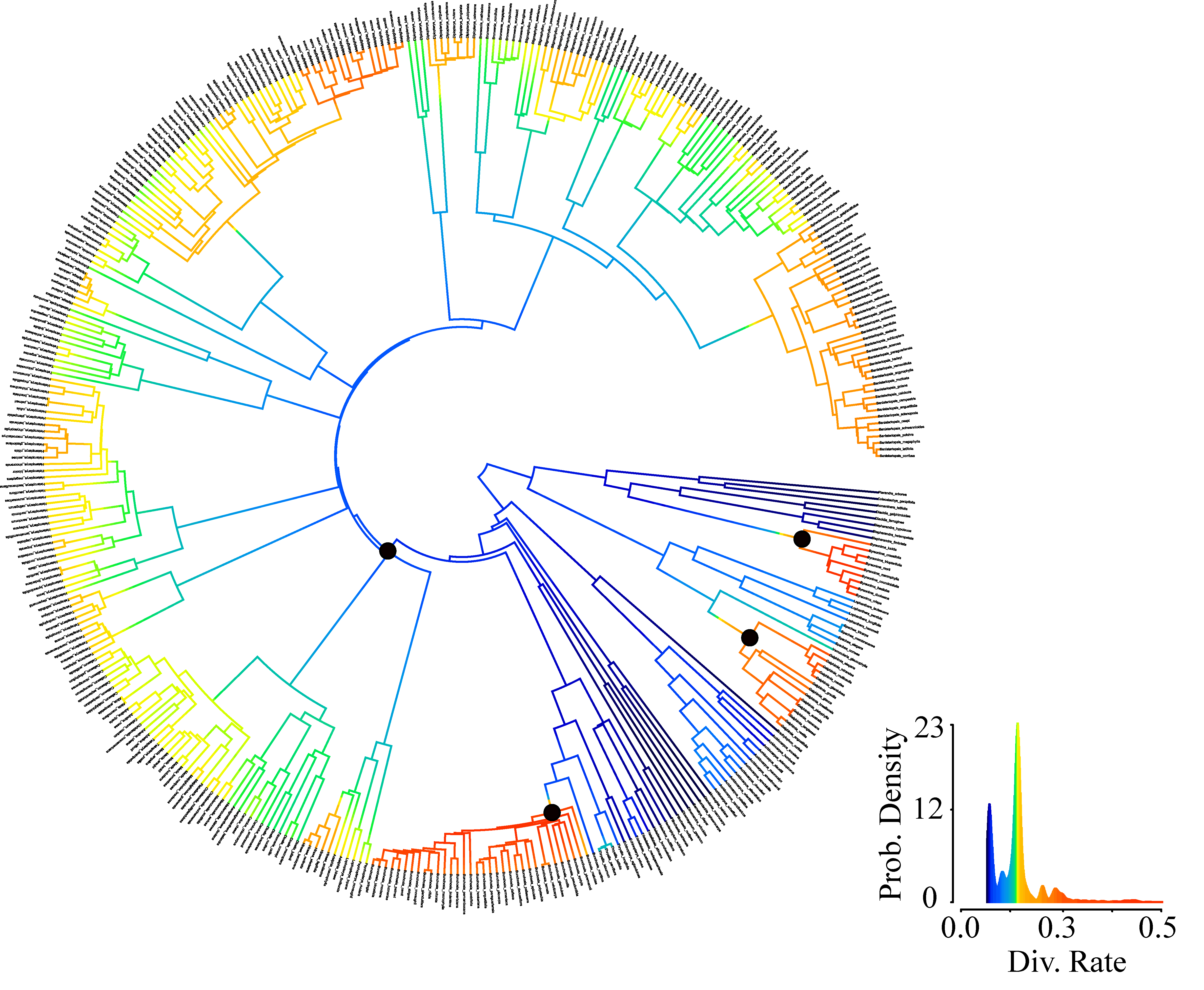

Supplement: Supplementary file 7 [file Image6.TIF]

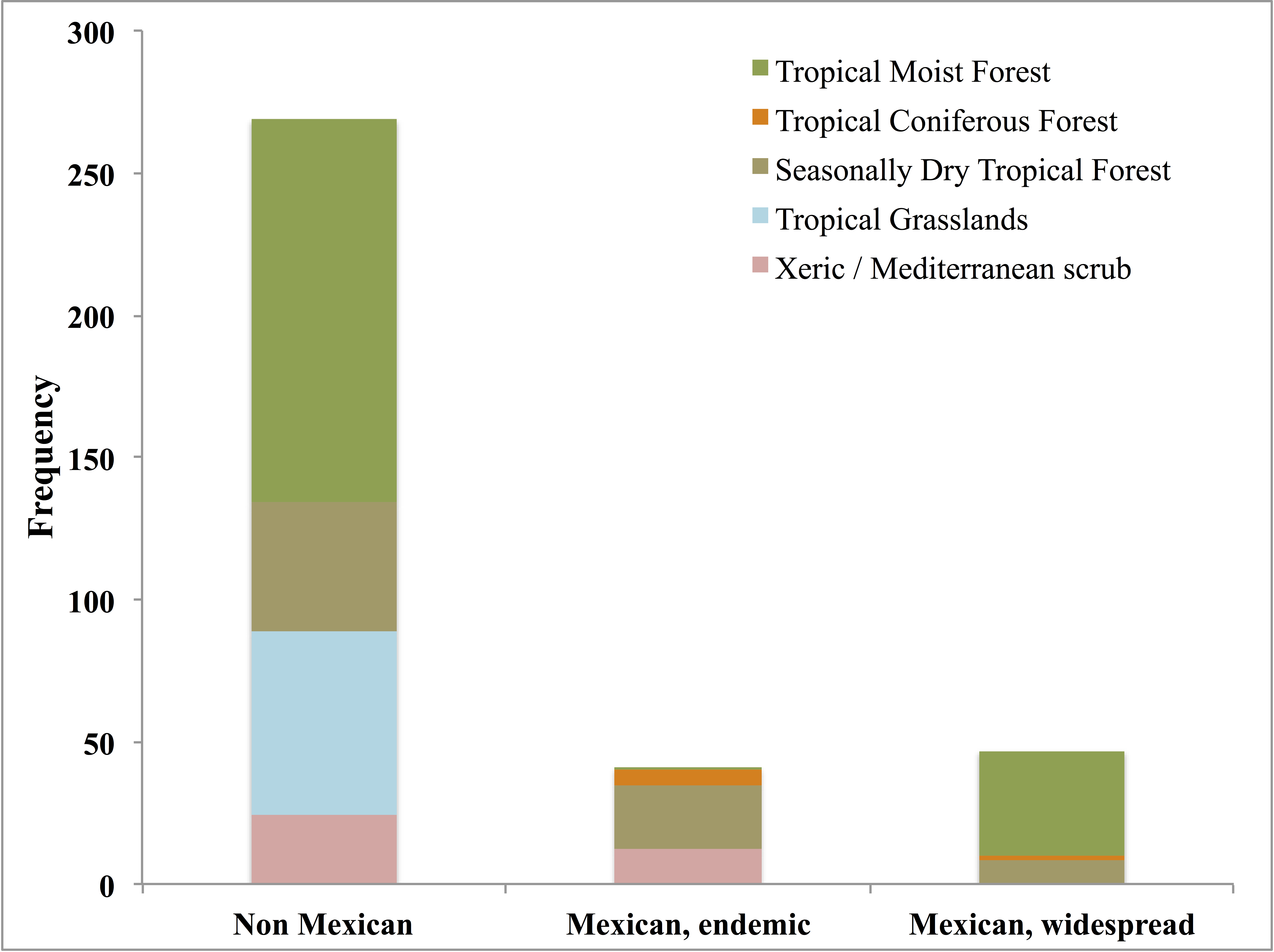

Supplement: Supplementary file 8 [file Image7.TIF]
